# Real-Time Detection and Prediction-Aided Dynamic Location Area Design for High-Mobility Users Based on LEO Satellites

**DOI:** 10.3390/s26175624

**Published:** 2026-09-04

**Authors:** An Chang, Xiaojin Ding, Gengxin Zhang

**Affiliations:** Telecommunication and Networks National Engineering Research Center, Nanjing University of Posts and Telecommunications, Nanjing 210003, China; 1224013814@njupt.edu.cn (A.C.);

**Keywords:** LEO satellite network, high-mobility user, coverage analysis, sensing and prediction fusion, dynamic location area design

## Abstract

In multi-beam low-Earth-orbit (LEO) satellite communication networks, high-mobility aerial users, such as unmanned aerial vehicles (UAVs), high-speed aircraft, and near-space vehicles, may traverse multiple satellite beams within a short period of time. When the precise position of a target user is not continuously available to the network, the network needs to determine the set of beams in which the user is likely to be located when a paging request arrives. The corresponding communication satellites then transmit paging messages within these candidate beams to reach the target user. If the selected beam set does not cover the user’s actual position, the paging attempt fails; however, excessively enlarging the paging region or frequently updating the user’s location information introduces additional signaling and management overhead. Therefore, the key problem is to construct an accurate and adaptive paging region under the joint mobility of the user and LEO satellite beams. To address this problem, this paper proposes a network-side sensing- and prediction-aided dynamic location-area management method for high-mobility users. First, based on a three-stage motion model of high-mobility users, LEO satellite ephemeris information, and beam coverage parameters, the coverage performance during the whole flight process of high-mobility users is analyzed. Second, a high-mobility user state prediction mechanism integrating a three-stage motion model and square-root cubature Kalman filtering (TSM-SRCKF) is proposed. This mechanism can adaptively adjust the weights of different motion models according to the current motion state of the high-mobility user and suppress the influence of abnormal measurements during the measurement update process, thereby obtaining more reliable position prediction results and error covariance information. Finally, a TSM-SRCKF-aided dynamic location-area management method is proposed. Simulation results show that the root-mean-square error of the high-mobility user position under the proposed mechanism is only 23.3% of that of the comparison mechanism. Compared with the traditional velocity-based dynamic location area design method, the proposed method improves the paging success probability by about 60.1% and reduces the cumulative total management overhead by about 73%.

## 1. Introduction

With the continuous development of unmanned aerial vehicle (UAV) technology, high-speed aerial platforms, and near-space vehicles [1,2], the need for communication support and position management for high-mobility users has become increasingly prominent. Traditional airborne user services primarily rely on ground networks or high-orbit satellite systems for auxiliary support. In areas such as the open sea, mountainous regions, deserts, and high-altitude flight routes, ground networks struggle to provide continuous and reliable service support to high-mobility users [3]. Although geostationary satellites can provide service to these areas [4], their high orbital altitude results in long link propagation distances, leading to significant transmission delays and path loss. Consequently, they face certain limitations when addressing the needs of high-speed, small-form-factor terminals and low-latency services. In contrast, LEO satellite networks feature low orbital altitudes, low transmission latency, and low link loss, enabling them to provide more flexible and continuous services to high-mobility users [5]. Therefore, researching location-management mechanisms that integrate LEO satellite networks with high-mobility users holds significant theoretical and practical value.

Location management for high-mobility users primarily involves two aspects: estimating the user’s motion state and designing location areas. For high-mobility users, their position, velocity, and acceleration change rapidly over time. If the network relies on users to actively report their own positions, it would require high-frequency location updates to maintain accurate location-management information, which would significantly increase satellite-to-ground signaling overhead and the communication burden on the user side. At the same time, in LEO satellite networks, the positions of both satellites and high-mobility users change rapidly. User-initiated position reporting may be affected by factors such as link interruptions, frequent handoffs, and delays, making it difficult to ensure continuous and reliable transmission. Furthermore, high-mobility users may sometimes need to conceal their location to prevent others from knowing it, and since uploaded position information is easily intercepted, this paper focuses on scenarios where high-mobility users do not actively upload their own position information. Motion state estimation involves using satellite sensing measurement data to estimate a user’s position, velocity, acceleration, and other states, thereby providing reliable positional prior information for subsequent paging [6,7,8].

Location-area design involves determining the spatial range within which a user is likely to be located based on the results of motion state estimation, and using this to select the set of satellite beams required for paging. When paging is required, the network side determines the paging-beam set based on the latest user-state estimate and delivers it to the corresponding communication satellites, which then transmit paging messages within the selected beams.

Since the motion states of both high-mobility users and LEO satellites change rapidly, and beam coverage areas continuously vary with satellite motion, the design of the location area directly affects paging success probability and location-management overhead: if the location area is too small, the user may have already left the predicted coverage area, resulting in paging failure; if the location area is too large, more satellite beams will need to participate in paging, leading to higher paging signaling and management overhead. Traditional fixed location-area management methods that design dynamic location areas based solely on user velocity and position [9] struggle to simultaneously ensure both paging success probability and low management overhead. Furthermore, traditional methods mainly relied on two-dimensional ground latitude and longitude information, typically considering only the user’s range of motion at their ground-projected position while ignoring the impact of altitude changes on satellite coverage. For ground-based mobile users, two-dimensional coverage cells can adequately describe the areas where users are likely to be located; however, for high-mobility users such as unmanned aerial vehicles (UAVs) and high-speed aircraft, altitude changes are significant during flight, and users are no longer within the ground coverage plane. In such cases, continuing to use two-dimensional ground coverage cells for coverage determination may lead to inaccurate estimates of beam coverage ranges, which, in turn, affects the selection of candidate beams and the level of management overhead. Therefore, the design of dynamic location areas for high-mobility users must expand from the traditional two-dimensional plane to three-dimensional space. This requires incorporating user-state predictions and their associated uncertainties to adaptively adjust the scope of the location areas, ensuring that the paging area covers all possible user locations while avoiding excessively large, ineffective search areas [10,11].

Recent studies on mobility and location management in LEO satellite networks have mainly focused on location-area design, mobility signaling optimization, and satellite-assisted localization. P. Du et al. proposed a distributed location-management scheme to balance location-update and paging overhead [12]. Alsaeedy and Chong highlighted signaling overhead as an important challenge in NTN mobility management [13]. You et al. investigated integrated communications and localization in LEO satellite systems [14]. However, these studies mainly address location management, signaling optimization, or localization separately. In contrast, this work jointly considers multi-satellite sensing, high-mobility user state prediction, prediction uncertainty, and time-varying beam geometry for dynamic location-area construction.

To address the above issues, this paper proposes a method for designing dynamic location areas for high-mobility users based on real-time detection and prediction using LEO satellites. First, to address the rapidly changing visibility and beam coverage relationships between satellites and high-mobility users during flight, a three-stage motion model for high-mobility users is established. By combining LEO satellite ephemeris information and beam coverage parameters, this model analyzes satellite visibility and beam coverage throughout the entire flight process. Second, to improve the accuracy of position prediction for high-mobility users, a state estimation method is designed that integrates the three-stage motion model with a square-root cubature Kalman filter. This method uses LEO satellite ranging and Doppler measurements to jointly estimate the user’s position, velocity, acceleration, and ranging bias, while adaptively adjusting the weights of different motion models based on the high-mobility user’s current motion state. Finally, based on the state prediction results and their error covariance, a dynamic location area construction method is proposed. This method maps the uncertainty in user position prediction onto a set of satellite beams, thereby determining the range of candidate beams required for paging and achieving a balance between paging reliability and management overhead. The main research work and contributions of this paper are as follows:

(1) A method for analyzing the coverage performance of LEO satellites for high-mobility users is proposed. For the three flight phases of a high-mobility user, ascent, cruise, and descent, a three-dimensional user motion trajectory model is established. By combining LEO satellite ephemeris information and beam coverage parameters, the set of observation satellites, beam coverage relationships, and coverage variation patterns throughout the entire flight process are analyzed.
(2) A state prediction method based on the three-stage motion model and square-root cubature Kalman filtering (TSM-SRCKF) is proposed for high-mobility users, based on LEO satellite ranging and Doppler measurements. This method incorporates both a constant-velocity (CV) model and a constant-acceleration (CA) model, as well as adaptively adjusting the weights of each model based on current measurement residuals. Furthermore, by integrating the square-root cubature Kalman filter, the method suppresses the impact of outlier observations on estimation results during the updating process of nonlinear ranging and Doppler measurements, to jointly estimate the user’s position, velocity, acceleration, and ranging bias, thereby yielding more reliable position prediction results and error covariance information.
(3) A TSM-SRCKF-aided dynamic location-area management (TSM-SRCKF-DLAM) method is proposed. Specifically, the user’s predicted position is used as the center of the dynamic location area; the uncertainty of the user’s future possible positions is characterized using the position error covariance; and an uncertainty region is constructed along the principal direction of the covariance. Subsequently, this uncertainty region is mapped onto the beam coverage relationship of LEO satellites to obtain a set of candidate beams along the principal direction, and the range of the dynamic location area is further determined based on the maximum distance from the candidate-beam centers to the location-area center and the corresponding effective beam radius at the user altitude. This method can adaptively adjust the size of the location area based on user prediction errors, thereby avoiding paging failures or redundant paging caused by traditional fixed location areas or those designed solely based on velocity.
(4) The effectiveness of the proposed method is verified through simulation experiments. The simulation results show that the proposed state estimation method can effectively reduce the position prediction error for high-mobility users, providing more accurate state and covariance information for dynamic location area design. Compared to traditional location area design methods based on user velocity, the method designed in this paper can significantly improve paging success probability while reducing the average number of paging beams and cumulative location-management overhead, demonstrating that the proposed method is better suited for mobility management scenarios involving high-mobility users in LEO satellite networks.

## 2. System Models and Coverage Analysis

### 2.1. System Model

Throughout this paper, t denotes the discrete time index corresponding to a sampling instant, i denotes the satellite index, j denotes the spot-beam index within a satellite, q and o denote motion-model indices, g denotes the sampling-point index on the principal-direction uncertainty line segment and p denotes the paging-attempt index.

**Figure 1:**
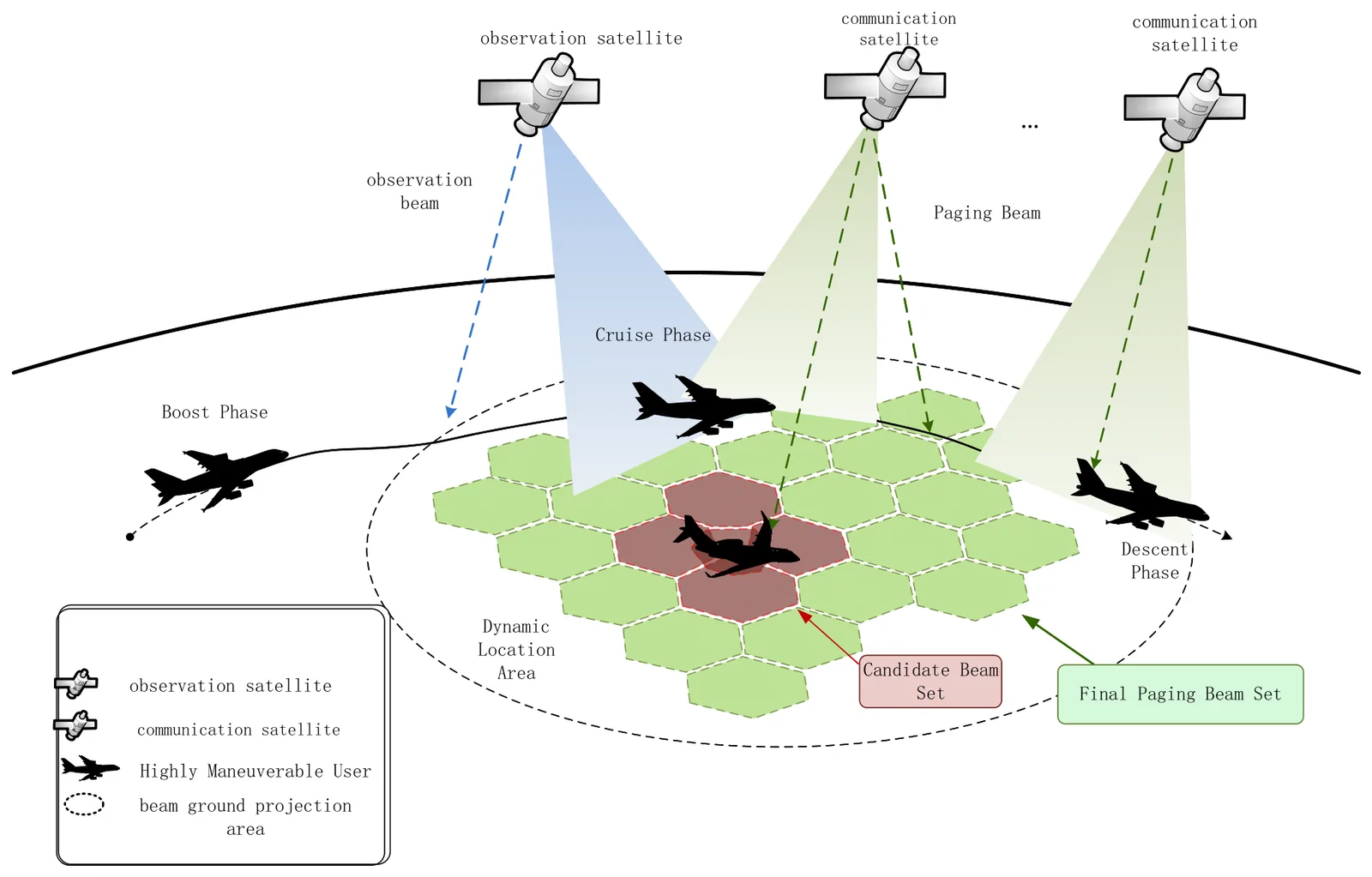
LEO-aided high-mobility user scenario with observation and communication satellites.

The scenario considered in this paper is shown in Figure 1, consisting of the spatial segment and the user segment. In modeling the spatial segment, this paper uses an LEO satellite constellation as the service network. The number of orbital planes is L, and the number of satellites in each orbital plane is G. The radius of the Earth is $R_{e}$, the orbital altitude of the satellites is $h_{s}$, and the orbital radius of the satellites can be expressed as $R_{s}=R_{e}+h_{s}$. At a given time t, the position vector of satellite i can be expressed as $r_{s,i}(t)={\left[x_{s,i}(t),\;y_{s,i}(t),\;z_{s,i}(t)\right]}^{T}$. Here, $x_{s,i}(t)$, $y_{s,i}(t)$, and $z_{s,i}(t)$ represent its three-dimensional coordinate components, respectively. As the satellite’s position changes over time, the corresponding latitude and longitude of the point of interest also change; therefore, the beam position is not fixed but is updated in real time as the satellite moves.

The user is modeled as a high-speed and highly dynamic aerial user. The initial user position is set as ($\varphi_{u,0}$, $\lambda_{u,0}$, $h_{u,0}$), and the user’s final position is set as ($\varphi_{u,f}$, $\lambda_{u,f}$, $h_{u,f}$). The user flies along a three-stage trajectory consisting of ascent, cruise and descent phases [11].

The user position vector is defined as $r_{u}(t)={\left[x_{u}(t),\;y_{u}(t),\;z_{u}(t)\right]}^{T}$, representing the three-dimensional position of the user in the ECEF coordinate system. The velocity vector is $v_{u}(t)={\left[v_{x}(t),\;v_{y}(t),\;v_{z}(t)\right]}^{T}$, and the acceleration vector is $a_{u}(t)={\left[a_{x}(t),\;a_{y}(t),\;a_{z}(t)\right]}^{T}$. The maximum flight speed of the user is denoted by $v_{u,\max}$, the acceleration in the ascent phase by $a_{u,\mathrm{up}}$, the acceleration in the descent phase by $a_{u,\mathrm{down}}$, and the cruising altitude by $h_{u,c}$. The durations of the ascent, cruise, and descent phases are denoted by $T_{up}$, $T_{c}$, and $T_{down}$, respectively. The total trajectory length is determined by the great-circle distance between the starting point and the destination, and the duration of each phase is then calculated according to the segmented velocity and acceleration relationships, thereby forming a parameterized three-stage motion model for high-mobility users.

### 2.2. Coverage Analysis

In an LEO satellite network, the position of a high-mobility user changes rapidly over time, while the coverage areas of satellite beams also vary continuously with satellite motion. A beam coverage analysis is used to characterize the time-varying service relationship between the high-mobility user and the LEO satellite network. Specifically, it determines whether the user can be effectively covered by at least one communication beam at a given time and establishes the geometric relationship between the user position and the available satellite beams. This coverage relationship provides the basis for subsequent dynamic location-area construction and paging-beam selection.

Meanwhile, observation satellites acquire ranging and Doppler measurements of the high-mobility user from the received sensing signals, where the ranging measurement characterizes the distance between the satellite and the user, and the Doppler measurement characterizes their relative radial velocity along the line-of-sight direction. Measurements from multiple observation satellites are then used to estimate the user state and predict its future position, as described in Section 3. The predicted position and its uncertainty are subsequently combined with the beam coverage relationship to determine the candidate beams in which the user may be located.

Assume that there are $N_{s}$ satellites in the LEO satellite constellation, and each satellite generates $N_{b}$ beams around its subsatellite point. The beam centers are arranged in a hexagonal pattern and move with the satellite motion. The latitude and longitude of the subsatellite point of the i-th satellite at time t are denoted as $\left(\varphi_{s,i}(t),\;\lambda_{s,i}(t)\right)$. The geodetic position vector of the high-mobility user at time t is expressed as $p_{u}(t)=\left[\varphi_{u}(t),\lambda_{u}(t),h_{u}(t)\right]$, where $\varphi_{u}(t)$, $\lambda_{u}(t)$ and $h_{u}(t)$ denote the latitude, longitude, and altitude of the user, respectively. The user altitude $h_{u}(t)$ is not fixed, varying continuously during the flight process. For a satellite beam with a fixed beamwidth, when the user altitude increases, the user becomes closer to the satellite, and the cross-sectional radius of the beam on the altitude plane where the user is located becomes smaller than its projection radius on the ground. Therefore, coverage analysis cannot simply use a fixed ground beam radius. Instead, the beam radius should be corrected according to the user altitude. At the user altitude $h_{u}(t)$, the center of beam j of satellite i is denoted as $p_{b,i,j}^{h}(t)=\left[\varphi_{b,i,j}^{h}(t),\lambda_{b,i,j}^{h}(t),h_{u}(t)\right]$, where the superscript h indicates that the corresponding beam geometry is evaluated on the horizontal plane at the user’s current altitude.

Assume that the altitude of satellite i at time t is $H_{s,i}(t)$ and that the beam radius on the ground is $R_{b}$. Then, the beam radius on the altitude plane where the user is located at time t can be expressed as [15]:

(1) $$R_{\mathrm{eff},i}(t)=R_{b}\frac{H_{s,i}(t)-h_{u}(t)}{H_{s,i}(t)}$$

As the user’s flight altitude increases, the coverage area of the satellite beam on the user’s altitude plane shrinks, and the coverage determination condition becomes stricter.

If the following condition is satisfied, $d_{u,i,j}^{h}(t)\leq R_{\mathrm{eff},i}(t)$, beam j of satellite i is considered able to cover the user at time t. Here, $d_{u,i,j}^{h}(t)$ denotes the distance between the user at altitude $h_{u}(t)$ and the beam center of the current satellite at the same altitude $h_{u}(t)$. If at least one beam of a satellite satisfies the above condition, the satellite is considered able to serve the user at the current time. If at least one satellite in the entire constellation can serve the user, the user is considered as being in the coverage state at that time. Conversely, if none of the beams across all satellites can cover the user, then the user is considered as being in an out-of-coverage state at that time.

After completing the coverage judgment for all sampling instants, the system coverage probability is defined as:

(2) $$P_{\mathrm{cov}}=\frac{N_{\mathrm{cov}}}{K}$$

where $N_{\mathrm{cov}}$ denotes the number of sampling instants at which the user is effectively covered, and K denotes the total number of sampling instants along the trajectory.

**Figure 2:**
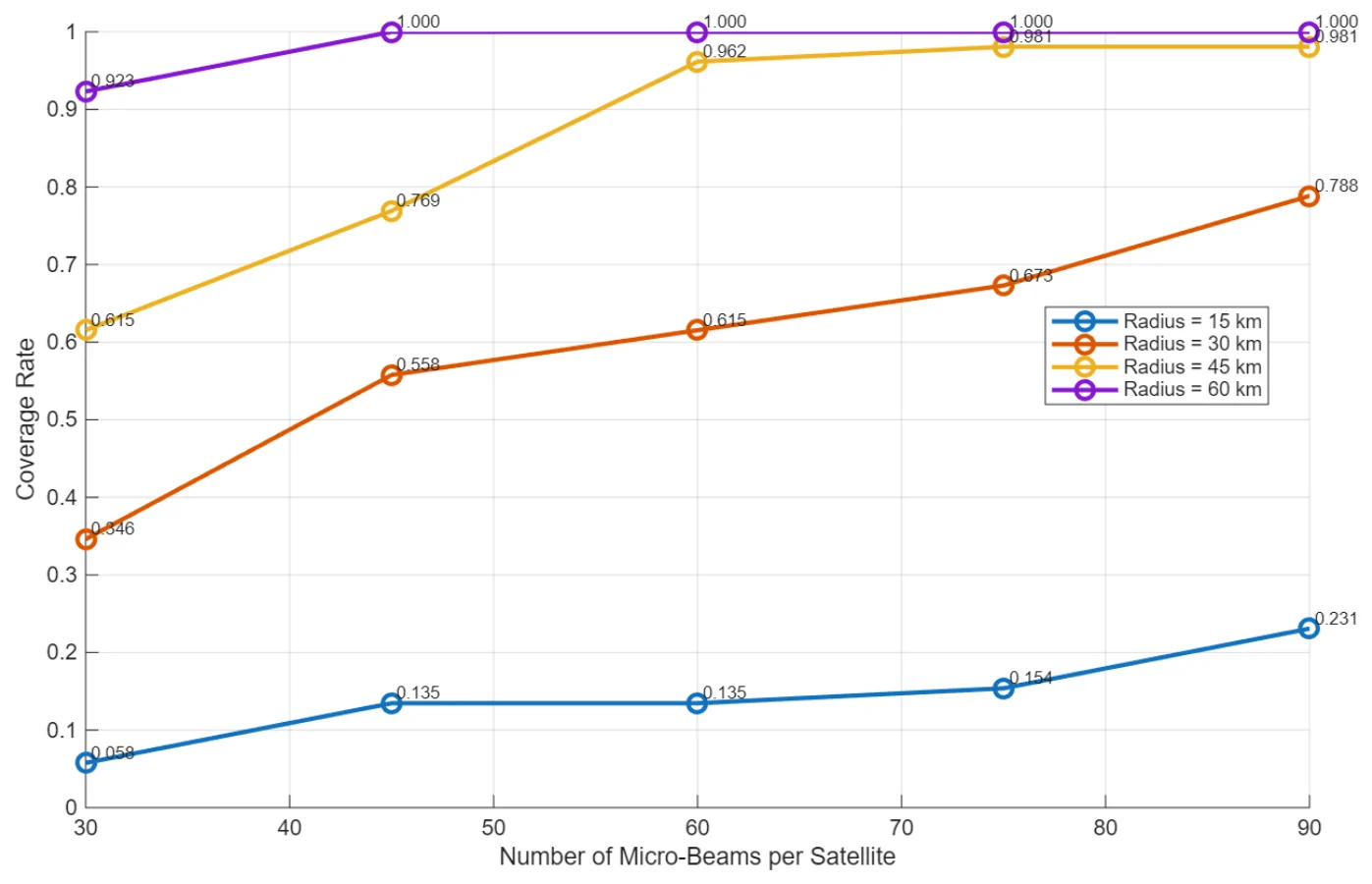
Coverage analysis under joint configurations of different beam numbers and beam radii.

Therefore, coverage analysis jointly considers the effects of the number of beams, beam radius, and user altitude. The number of beams hit by the user, the number of observation satellites, and the distribution of serving beams are counted at each time instant. Finally, the above process is repeated under different beam-number and beam-radius configurations, and the coverage comparison results are obtained as shown in Figure 2.

Considering both coverage probability and beam resource overhead, when the beam radius is 45 km and the number of beams is 60, the coverage probability reaches 0.962, achieving near-continuous coverage with relatively low beam overhead. If full-trajectory coverage is required, a beam radius of 60 km with 45 beams is sufficient to achieve a coverage probability of 1.000.

## 3. High-Mobility User State Estimation Method Based on Detection and Prediction Fusion

To satisfy the coverage requirements, the system further introduces a detection and prediction algorithm based on satellite signals. Since continuous high-frequency detection increases power consumption and resource overhead, and satellite sensing measurements are affected by noise and ranging bias, state estimation is used to obtain the current motion-state estimate of the high-mobility user, while prediction is used to infer the possible position at a future paging instant. This provides continuous and reliable state estimation and uncertainty information for dynamic location area design. Let the satellite signal transmit power be $P_{tx}$, the operating frequency be $f_{c}$, the carrier wavelength be λ, the number of coherent accumulated pulses be $N_{p}$, the pulse width be $T_{p}$, the detection coherent integration time be $T_{\mathrm{coh}}$, and the prediction time be $T_{\mathrm{idle}}$.

Since a single ranging measurement only constrains the distance between an observation satellite and the high-mobility user, it cannot uniquely determine the user’s three-dimensional position. Geometrically, a single-satellite ranging measurement constrains the possible user position to a spherical surface centered at the satellite, whereas observations from multiple satellites provide additional spatial constraints. Therefore, reliable estimation of the three-dimensional motion state of a high-mobility user requires the fusion of ranging and Doppler measurements acquired by multiple visible observation satellites over successive sampling epochs.

In this study, the observation satellites employ active radar sensing with linear-frequency-modulated (LFM) pulses rather than communication-signal-based ranging. The LFM waveform is characterized by the pulse width $T_{p}$ and bandwidth $B_{LFM}$. The reflected echoes are processed through matched filtering and coherent processing to obtain the ranging and Doppler-derived radial-velocity measurements. To relate the physical sensing parameters to the statistical measurement model, the effective received echo signal-to-noise ratio (SNR) of the i-th observation satellite at sampling instant t is modeled as

(3) $$\gamma_{i,t}=N_{p}\frac{P_{\mathrm{tx}}G_{\mathrm{tx}}G_{\mathrm{rx}}\lambda^{2}\sigma_{\mathrm{RCS},t}}{{\left(4\pi \right)}^{3}{\left\|r_{u}(t)-r_{s,i}(t)\right\|}^{4}k_{B}T_{0}F_{n}B_{n}L_{\mathrm{sys}}}$$

where $p_{tx}$ is the radar transmit power; $G_{tx}$ and $G_{rx}$ are the transmit and receive antenna gains, respectively; λ is the carrier wavelength; $\sigma_{\mathrm{RCS},t}$ denotes the instantaneous radar cross-section of the target user at sampling instant t; $k_{B}$ is the Boltzmann constant; $T_{0}$ is the reference noise temperature; $F_{n}$ is the receiver noise factor; $B_{n}$ is the equivalent noise bandwidth; $L_{\mathrm{sys}}$ is the total system loss; $N_{p}$ is the number of coherently processed pulses; $r_{u,t}$ and $r_{s,i,t}$ denote the position vectors of the target user and the i-th observation satellite at sampling instant t, respectively; and $\gamma_{i,t}$ denotes the effective received echo SNR after coherent processing.

Accordingly, the standard deviations of the ranging and Doppler-derived radial-velocity measurements are modeled as

(4) $$\sigma_{R,i,t}=\frac{c_{0}}{2B_{\mathrm{LFM}}\sqrt{2\gamma_{i,t}}},\;\;\sigma_{v,i,t}=\frac{\lambda }{4\pi T_{\mathrm{coh}}\sqrt{2\gamma_{i,t}}}$$

where $c_{0}$ is the speed of light, $B_{LFM}$ is the LFM bandwidth, and $T_{coh}$ is the coherent processing interval. Therefore, the corresponding measurement noises satisfy

(5) $$w_{R,i,t}\sim N\left(0,\sigma_{R,i,t}^{2}\right),\;\;w_{v,i,t}\sim N\left(0,\sigma_{v,i,t}^{2}\right)$$

where $\sigma_{R,i,t}^{2}$ and $\sigma_{v,i,t}^{2}$ are the ranging and Doppler measurement-noise variances, respectively.

For each observation satellite, the ranging measurement is derived from the propagation delay of the received sensing echo and characterizes the instantaneous slant range between the satellite and the target user. The Doppler measurement is obtained by estimating the Doppler frequency shift induced by the relative motion between the satellite and the user and is further represented as the relative radial velocity projected onto the satellite–user line-of-sight direction. In the considered sensing model, both ranging and Doppler measurements are extracted from the received sensing echoes through coherent processing rather than through communication-payload decoding. Accordingly, these two types of measurements provide complementary constraints on the user’s position and velocity states and are jointly incorporated into the subsequent state-estimation process.

This study focuses on the state estimation and location management of a designated high-mobility user. Before the ranging and Doppler measurements are provided to the proposed state estimator, target detection and measurement-to-target association are assumed to have been completed by the sensing front end. In scenarios involving multiple targets, range–Doppler processing, together with conventional target-tracking and data-association procedures, can be used to distinguish measurements associated with different targets and associate them with the corresponding target tracks. Accordingly, the subsequent state-estimation process uses only the ranging and Doppler measurements associated with the target user of interest.

The observation satellites are assumed to be synchronized to a common network time and frequency reference before cooperative sensing. Satellite-dependent timing offsets are calibrated before measurement fusion, while the remaining equivalent ranging bias $b_{R,t}$ is jointly estimated with the user motion state. Residual carrier-frequency offsets are assumed to be compensated before Doppler estimation and are, therefore, not explicitly introduced as additional state variables. Since this work focuses on post-detection state estimation and dynamic location-area management, the ranging and Doppler measurements are conditioned on successful target detection and association; missed detections, false alarms, and radar detector optimization are not explicitly modeled.

Let $M_{t}$ denote the number of selected visible observation satellites at sampling instant t, and let $M_{\max}$ denote the maximum number of observation satellites used for one filtering epoch. In this study,

(6) $$0\leq M_{t}\leq M_{\max},\;\;M_{\max}=4$$

When $M_{t}\geq 1$, the available measurements are sequentially used for recursive state updating, whereas when $M_{t}=0$, only state prediction is performed. A single visible satellite, therefore, allows a recursive measurement update, but does not imply an instantaneous standalone three-dimensional positioning solution.

At each sampling instant, the selected observation satellites provide ranging and Doppler measurements for the subsequent state-estimation process.

Let $r_{s,i}(t)$ denote the position of satellite i at time t, and let $z_{R,i,t}$ denote the ranging measurement obtained by satellite i at time t [16], where $1\leq i\leq M_{t}$. Then, $z_{R,i,t}$ can be expressed as

(7) $$z_{R,i,t}=\left\|r_{u}(t)-r_{s,i}(t)\right\|+b_{R,t}+w_{R,i,t}$$

where $r_{u,t}$ denotes the position vector of the high-mobility user at sampling instant t, $b_{R,t}$ is the ranging bias, and $w_{R,i,t}$ denotes the ranging measurement noise of the i-th observation satellite at sampling instant t. Based on $z_{R,i,t}$, $r_{u,t}$ and $b_{R,t}$ can be further expressed as:

(8) $$\left(r_{u}(t),\;{\hat{b}}_{R,t}\right)=\arg\underset{r,\;b_{R}}{\min}\sum_{i=1}^{M_{t}}{\left(z_{R,i,t}-\left\|r-r_{s,i}(t)\right\|-b_{R}\right)}^{2}$$

where r and $b_{R}$ denote the candidate user-position vector and ranging-bias variable, respectively, in the least-squares optimization. By minimizing the objective function in Equation (8), the optimal solutions of r and $b_{R}$ are obtained as $r_{u,t}$ and ${\hat{b}}_{R,t}$, respectively. In addition, let $z_{v,i,t}$ denote the Doppler measurement obtained by satellite i at time t [16]:

(9) $$z_{v,i,t}={\hat{u}}_{i,t}^{T}\left(v_{u}(t)-v_{s,i}(t)\right)+w_{v,i,t}$$

where $v_{s,i}(t)$ denotes the velocity vector of the i-th observation satellite at sampling instant t, $v_{u}(t)$ denotes the velocity vector of the high-mobility user at sampling instant t, $w_{v,i,t}$ denotes the Doppler measurement noise, and ${\hat{u}}_{i,t}$ denotes the unit line-of-sight vector from the i-th observation satellite to the high-mobility user. It is given by

(10) $${\hat{u}}_{i,t}=\frac{r_{u}(t)-r_{s,i}(t)}{\left\|r_{u}(t)-r_{s,i}(t)\right\|}$$

In addition, based on $z_{v,i,t}$ and ${\hat{u}}_{i,t}$, $v_{u}(t)$ can be obtained as

(11) $$v_{u}(t)=\arg\underset{v}{\min}\sum_{i=1}^{M_{t}}{\left[z_{v,i,t}-{\hat{u}}_{i,t}^{T}\left(v-v_{s,i}(t)\right)\right]}^{2}$$

where v denotes the candidate user velocity vector in the least-squares optimization. By substituting $z_{v,i,t}$, ${\hat{u}}_{i,t}$ and $v_{s,i}(t)$ into (11), the objective of (11) is to find the value of v that minimizes the summation on the right-hand side. The optimal solution can then be obtained as $v_{u}(t)$. In addition, the state vector of the high-mobility user is defined as $x_{t}={\left[r_{u}(t),v_{u}(t),a_{u}(t),b_{R,t}\right]}^{T}$. In the prediction stage, the prior predicted state ${\hat{x}}_{t-1|t-1}$ and the predicted covariance $P_{t-1|t-1}$ at the current time are obtained according to the posterior state estimate ${\hat{x}}_{t|t-1}$, the posterior covariance $P_{t|t-1}$ at the previous time, and the motion model. Then, the satellite ranging and Doppler measurements at the current time are used to correct the predicted state, yielding the posterior state estimate ${\hat{x}}_{t|t}$ and the posterior covariance $P_{t|t}$.

Since the motion characteristics of high-mobility users vary significantly across different flight phases, using only a constant-acceleration (CA) model or a constant-velocity (CV) model may lead to model mismatch. If only the CV model is used, it may fail to track the user during highly maneuvering phases. If only the CA model is used, the estimation may fluctuate during the steady cruising phase. During transitions between maneuvering phases, a single model struggles to balance estimation stability and tracking speed. The interacting multiple model (IMM) method improves adaptability to complex maneuvering states by performing parallel estimation with multiple motion models and fusing their model probabilities [17]. In this paper, the CV and CA models are adopted [18]. Let the posterior state estimate, posterior covariance, and model probability of model q at time t−1 be ${\hat{x}}_{t-1|t-1}^{q}$, $P_{t-1|t-1}^{q}$ and $\mu_{t-1}^{q}$. The model transition probability matrix is denoted as $\Pi =[\pi_{oq}]$, where $\pi_{oq}$ represents the probability of transition from model o to model q. The normalization factor of model q is calculated as [18]:

(12) $$c_{q}=\sum_{o=1}^{n}\pi_{oq}\mu_{t-1}^{o}$$

Then, the mixing probability is calculated as [18]

(13) $$\mu_{o|q}=\frac{\pi_{oq}\mu_{t-1}^{o}}{c_{q}}$$

where $\mu_{o|q}$ denotes the conditional mixing probability used to weight the contribution of motion sub-model O to the mixed initial state of motion sub-model q. Thus, the mixed initial state of model q is obtained as [16]:

(14) $${\bar{x}}_{t-1|t-1}^{q}=\sum_{o=1}^{n}\mu_{o|q}{\hat{x}}_{t-1|t-1}^{o}$$

where n is the number of models.

The corresponding mixed covariance is given by:

(15) $${\bar{P}}_{t-1|t-1}^{q}=\sum_{o=1}^{n}\mu_{oq}\left[P_{t-1|t-1}^{o}+\left({\hat{x}}_{t-1|t-1}^{o}-{\bar{x}}_{t-1|t-1}^{q}\right){\left({\hat{x}}_{t-1|t-1}^{o}-{\bar{x}}_{t-1|t-1}^{q}\right)}^{T}\right]$$

Then, each sub-model performs prediction according to its own motion model, yielding:

(16) $${\hat{x}}_{t|t-1}^{q}=F^{q}{\bar{x}}_{t-1|t-1}^{q}$$

and

(17) $$P_{t|t-1}^{q}=F^{q}{\bar{P}}_{t-1|t-1}^{q}{\left(F^{q}\right)}^{T}+Q^{q}$$

where $F^{q}$ and $Q^{q}$ denote the state transition matrix and the process noise matrix corresponding to model q, respectively. The state transition matrix of the CA model is expressed as:

(18) $$F_{t}^{\mathrm{CA}}=\left[\begin{array}{ccc} I_{3} & \Delta tI_{3} & \frac{1}{2}\Delta t^{2}I_{3}\\ 0 & I_{3} & \Delta tI_{3}\\ 0 & 0 & I_{3} \end{array}\right]$$

where $I_{3}$ denotes the third-order identity matrix, and Δt denotes the sampling time interval between filtering epochs. In addition, the state transition matrix of the CV model is expressed as

(19) $$F_{t}^{\mathrm{CV}}=\left[\begin{array}{ccc} I_{3} & \Delta tI_{3} & \frac{1}{2}\Delta t^{2}I_{3}\\ 0 & I_{3} & \Delta tI_{3}\\ 0 & 0 & \rho_{a}I_{3} \end{array}\right]$$

where $\rho_{a}$ denotes the correlation coefficient between the acceleration at the current time and that at the previous time.

The process noise matrix $Q^{q}$ is given by:

(20) $$Q_{t}^{q}=\beta_{q}\left[\begin{array}{ccc} \frac{\Delta t^{5}}{20}I_{3} & \frac{\Delta t^{4}}{8}I_{3} & \frac{\Delta t^{3}}{6}I_{3}\\ \frac{\Delta t^{4}}{8}I_{3} & \frac{\Delta t^{3}}{3}I_{3} & \frac{\Delta t^{2}}{2}I_{3}\\ \frac{\Delta t^{3}}{6}I_{3} & \frac{\Delta t^{2}}{2}I_{3} & \Delta tI_{3} \end{array}\right]$$

where $\beta_{q}$ denotes the process-noise intensity coefficient of model q.

Within each state update interval, each motion sub-model predicts the user state according to its corresponding CV or CA state-transition model. Then, the observation satellites obtain ranging and Doppler measurements from the coherent detection results over $T_{\mathrm{coh}}$, and the prior predicted state is corrected using the measurement residuals. Since both the ranging and Doppler measurement functions are nonlinear, the square-root cubature Kalman filter is adopted in this paper to perform nonlinear measurement updates.

For the ranging measurement, when the user state is $x_{t}$, the theoretical range measurement that should be obtained by satellite i is given by:

(21) $$h_{R,i}(x_{t})=\left\|r_{u}(t)-r_{s,i}(t)\right\|+b_{R,t}$$

where $r_{u,t}$ denotes the position component in the user-state vector. In the actual filtering update process, the true user state $x_{t}$ is unknown. Therefore, the predicted state ${\hat{x}}_{t|t-1}^{q}$ of model q is substituted into the above equation to obtain the predicted range value. The difference between this predicted range and the actual ranging measurement $z_{R,i,t}$ forms the ranging residual. This residual is used to correct the predicted state. The difference between the actual ranging measurement and the predicted ranging measurement, denoted by $e_{R,i,t}^{q}$, can be expressed as

(22) $$e_{R,i,t}^{q}=z_{R,i,t}-h_{R,i}\left({\hat{x}}_{t|t-1}^{q}\right)$$

For the Doppler measurement, when the user state is $x_{t}$, the theoretical Doppler radial velocity that should be observed by satellite i is given by

(23) $$h_{v,i}(x_{t})={\hat{u}}_{i,t}^{T}\left(v_{u}(t)-v_{s,i}(t)\right)$$

where ${\hat{u}}_{i,t}^{T}$ denotes the transpose of the unit line-of-sight vector. It is used to take the inner product with the relative velocity, thereby projecting the three-dimensional relative velocity onto the line-of-sight direction between the satellite and the user. By substituting the predicted state ${\hat{x}}_{t|t-1}^{q}$ of model q into the Doppler measurement function, the predicted radial velocity is obtained. The difference between this predicted radial velocity and the actual measurement forms the Doppler residual, which is used to correct the predicted velocity and the related state components. In addition, the difference between the actual Doppler radial-velocity measurement and the predicted radial velocity, denoted by $e_{v,i,t}^{q}$, can be expressed as

(24) $$e_{v,i,t}^{q}=z_{v,i,t}-h_{v,i}\left({\hat{x}}_{t|t-1}^{q}\right)$$

When multiple observation satellites are available at the same time, the ranging and Doppler measurements of each satellite are sequentially fed into the filter for updating. Each update uses the current measurement residual to correct the predicted state so that the state estimate is progressively corrected using the available measurements.

In the specific update process, at each sampling epoch, the ranging measurements and Doppler measurements from the observation satellites are sequentially input into the square-root cubature Kalman filter. The ranging measurements are used to constrain the spatial distance between the user and the satellite, thereby correcting the predicted position and ranging bias. The Doppler measurements are used to constrain the relative radial velocity between the user and the satellite, thereby correcting the predicted velocity and acceleration components. When no visible observation satellite is available at the current epoch, the filter performs only state prediction according to the motion model and propagates the model weights through the model transition probabilities.

When visible observation satellites are available, the ranging and Doppler residuals are used as the inputs of the filtering update to correct the predicted state. The state update can be uniformly expressed as:

(25) $${\hat{x}}_{t|t}^{q}={\hat{x}}_{t|t-1}^{q}+K_{t}^{q}e_{t}^{q}$$

where ${\hat{x}}_{t|t}^{q}$ is the updated state after fusing the satellite sensing measurements, $K_{t}^{q}$ is the filter gain, and $e_{t}^{q}$ is the current ranging or Doppler measurement residual. When the ranging measurement of satellite i is input, $e_{t}^{q}=e_{R,i,t}^{q}$. When the Doppler measurement of satellite i is input, $e_{t}^{q}=e_{v,i,t}^{q}$.

Since satellite measurement accuracy is affected by factors such as the user’s attitude, RCS fluctuation, signal-to-noise ratio variation, and ranging bias, the ranging or Doppler measurements at some epochs may contain relatively large deviations. To improve the robustness of the filtering process against abnormal measurements, this paper adaptively adjusts the measurement update strength according to the consistency between the measurement residual and the innovation covariance. When the residual is small, it indicates that the current measurement matches the predicted state well, and the filter can normally use this measurement for correction. When the residual is large, it indicates that the current measurement may be affected by noise or echo fluctuation, and the filter reduces the influence of this measurement on the state update, thereby suppressing the estimation bias caused by abnormal measurements.

After each sub-model completes prediction and measurement correction, the likelihood of the model is calculated according to the measurement residual and innovation covariance under that model. During the model probability update, it is necessary to determine the matching degree between different motion models and the current satellite sensing measurements. For model q, the difference between its predicted observation and the actual measurement is first calculated. The combined residual of the ranging and Doppler measurements is denoted as $J_{t}^{q}$. The smaller the residual is, the closer the prediction result of the model is to the satellite sensing measurement, and the more reliable the model is. Meanwhile, the innovation covariance characterizes the uncertainty associated with the measurement residual. When the innovation covariance is large, even a relatively large residual does not necessarily indicate a model mismatch because the measurement noise or prediction uncertainty may also be large. Conversely, when the innovation covariance is small, a large residual is more likely to indicate that the motion model does not match the user’s current motion state. Therefore, the model likelihood can be expressed as

(26) $$\Lambda_{q}=\exp\left(-\frac{1}{2}J_{t}^{q}\right)$$

where $\Lambda_{q}$ denotes the likelihood of motion sub-model q given the current satellite sensing measurements. The measurement residual is jointly composed of the ranging residuals and Doppler residuals of all observation satellites at the current time. For simplicity, the combined measurement residual used for model likelihood calculation under model q is denoted as $J_{t}^{q}$. Here, $S_{R,i,t}^{q},S_{v,i,t}^{q}$ denote the innovation covariance corresponding to the ranging residual and the Doppler residual, respectively. $J_{t}^{q}$ can be given by

(27) $$J_{t}^{q}=\sum_{i=1}^{M}\left[\frac{{\left(e_{R,i,t}^{q}\right)}^{2}}{S_{R,i,t}^{q}}+\frac{{\left(e_{v,i,t}^{q}\right)}^{2}}{S_{v,i,t}^{q}}\right]$$

The model probability is updated as

(28) $$\mu_{t}^{q}=\frac{c_{q}\Lambda_{q}}{\sum_{l=1}^{n}c_{l}\Lambda_{l}}$$

where $c_{q}$ is the normalization factor obtained in the model interaction stage, and l is a dummy index used to sum over all motion sub-models. If a certain model can better explain the current satellite sensing measurements, its model probability will increase; otherwise, its model probability will decrease. In this way, the IMM method adaptively adjusts the probabilities of the CV and CA motion sub-models during the ascent, cruise, and descent phases.

Finally, the states of all sub-models are fused according to the model probabilities:

(29) $${\hat{x}}_{t|t}=\sum_{q=1}^{n}\mu_{t}^{q}{\hat{x}}_{t|t}^{q}$$

The overall covariance is given by

(30) $$P_{t|t}=\sum_{q=1}^{n}\mu_{t}^{q}\left[P_{t|t}^{q}+\left({\hat{x}}_{t|t}^{q}-{\hat{x}}_{t|t}\right){\left({\hat{x}}_{t|t}^{q}-{\hat{x}}_{t|t}\right)}^{T}\right]$$

The fused state can be written as

(31) $${\hat{x}}_{t|t}={\left[{\hat{r}}_{u,t|t}^{T},{\hat{v}}_{u,t|t}^{T},{\hat{a}}_{u,t|t}^{T},{\hat{b}}_{R,t|t}\right]}^{T}$$

where ${\hat{r}}_{u,t|t}$ is the user position estimate, ${\hat{v}}_{u,t|t}$ is the velocity estimate, ${\hat{a}}_{u,t|t}$ is the acceleration estimate, ${\hat{b}}_{R,t|t}$ is the ranging bias estimate, and $P_{t|t}$ is the state estimation covariance. The overall procedure of the high-mobility user state estimation method based on detection and prediction fusion is shown in Algorithm 1.


**Algorithm 1:** High-Mobility User State Estimation Algorithm Based on Detection and Prediction Fusion
**Input:**
Satellite ephemeris data; initial state of the high-mobility user $x_{0},$ initial covariance $p_{0},$ model transition probability matrix Π of the CV/CA motion-model set; ranging measurements $z_{R},$ Doppler measurements $z_{v},$ simulation sampling interval Δt, number of sampling instants K

**Initialization:**
$\mathrm{Initialize}\;\mathrm{the}\;\mathrm{state}\;x_{0}^{q},$ $\mathrm{covariance}\;P_{0}^{q},$ $\mathrm{and}\;\mathrm{model}\;\mathrm{probability}\;\mu_{0}^{q}$ of each motion sub-model in the CV/CA model set, where q=1,2…n and n denotes the number of sub-models.1

For t=1,2,…,K

2$\mathrm{Read}\;\mathrm{the}\;\mathrm{position}\;r_{s,i,t}$ of the satellite i at the current time and the prior trajectory information of the user, and select the observation satellites set according to the geometric visibility condition.3Obtain the ranging measurement from each visible observation satellite: $z_{R,i,t}=\left\|r_{u}(t)-r_{s,i}(t)\right\|+b_{R,t}+w_{R,i,t}$
4Obtain the Doppler/radial-velocity measurement from each visible observation satellite: $z_{v,i,t}={\hat{u}}_{i,t}^{T}\left(v_{u}(t)-v_{s,i}(t)\right)+w_{v,i,t}$
5$\mathrm{Calculate}\;\mathrm{the}\;\mathrm{mixing}\;\mathrm{probabilities}\;\mu_{o|q}$ according to the model transition probabilities and the model probabilities at the previous time instant. 6Perform state mixing for each motion sub-model in the CV/CA model set and obtain the mixed initial value ${\hat{x}}_{t-1|t-1}^{q}$ *and mixed covariance*
${\bar{P}}_{t-1|t-1}^{q}$ of sub-model q.7Perform state prediction under the CV model and the CA model, respectively, and obtain the predicted state ${\hat{x}}_{t|t-1}^{q}$ *and predicted covariance*
$P_{t|t-1}^{q}$ of each sub-model. 8Calculate the ranging and Doppler residuals:
$e_{R,t}^{q}=z_{R,i,t}-h_{R}\left({\hat{x}}_{t|t-1}^{q}\right),$ $e_{v,t}^{q}=z_{v,i,t}-h_{v}\left({\hat{x}}_{t|t-1}^{q}\right)$
9For each sub-model, perform a scalar square-root cubature Kalman filtering update based on the ranging measurement $z_{R,i,t}.$ The ranging residual is used to correct the predicted state, yielding the state and covariance after ranging update.10For each sub-model, perform a scalar square-root cubature Kalman filtering update based on the Doppler measurement $z_{v,i,t}.$ The Doppler residual is used to further correct the state, yielding the state and covariance after Doppler update.11Calculate the model likelihood according to the measurement residual and its covariance for each sub-model, and update the model probabilities $\mu_{t}^{q}$ of the CV and CA motion sub-models in combination with the model transition probabilities.12Fuse the states and covariances of all sub-models according to the updated model probabilities, and obtain the overall state estimate: ${\hat{x}}_{t|t}=\sum_{q=1}^{n}\mu_{t}^{q}{\hat{x}}_{t|t}^{q}$
13Extract the user position, velocity, acceleration, and ranging bias estimates from the overall state estimate ${\hat{x}}_{t|t}={\left[{\hat{r}}_{u,t|t}^{T},{\hat{v}}_{u,t|t}^{T},{\hat{a}}_{u,t|t}^{T},{\hat{b}}_{R,t|t}\right]}^{T}.$
14$\mathrm{Store}\;{\hat{r}}_{u,t|t},$ ${\hat{v}}_{u,t|t},$ ${\hat{a}}_{u,t|t},$ $\;{\hat{b}}_{R,t|t}$ $\mathrm{and}P_{t|t}.$
**Output**
$\mathrm{User}\;\mathrm{position}\;\mathrm{estimate}\;{\hat{r}}_{u,t|t},$ $\mathrm{user}\;\mathrm{velocity}\;\mathrm{estimate}\;{\hat{v}}_{u,t|t},$ $\mathrm{user}\;\mathrm{acceleration}\;\mathrm{estimate}\;{\hat{a}}_{u,t|t},$ $\mathrm{ranging}\;\mathrm{bias}\;\mathrm{estimate}\;{\hat{b}}_{R,t|t},$ $\mathrm{state}\;\mathrm{estimation}\;\mathrm{covariance}\;P_{t|t},$ 1≤t≤K.


## 4. Dynamic Location Area Design Method Based on User Position and Its Covariance

In LEO satellite networks, high-mobility users move rapidly and their trajectories are not fixed. Within a short period of time, they may cross multiple beam coverage areas. If fixed location-area management is adopted, an excessively small location area may easily lead to a paging failure, whereas an excessively large location area will increase the number of paging beams and raise system overhead. Therefore, a TSM-SRCKF-aided dynamic location-area management (TSM-SRCKF-DLAM) method is proposed, using the current user-state estimation results.

For a high-mobility user, traditional location-area management schemes, usually triggering a location update when the user leaves the registration area or when the movement distance exceeds a threshold, may introduce large signaling overhead in the presence of relatively frequent location updates. Thus, the proposed TSM-SRCKF-DLAM method further adopts a paging-triggered location-area delivery mechanism. During non-paging periods, the network side maintains the user-state estimation results, but does not continuously deliver the location area to the communication satellites. When a paging request occurs, the dynamic location area is constructed according to the latest state prediction and covariance, and the corresponding paging beam set is sent to the relevant communication satellites.

Under this mechanism, observation satellites and communication satellites perform different functions. The observation satellites acquire ranging and Doppler measurements and forward the corresponding measurement reports to the network-side state estimator, directly or through inter-satellite links when necessary. The network side performs TSM-SRCKF state estimation and maintains the latest user-state estimate and covariance. During non-paging periods, these state-estimation results are not continuously delivered to the communication satellites. When a paging request arrives, the network side constructs the dynamic location area according to the latest state estimate and covariance, determines the corresponding paging-beam set, and delivers it to the relevant communication satellites. The communication satellites then transmit paging messages within the designated beams.

In this paper, the dynamic location area is represented by a circular region. The center is the predicted value of the user position at the current time, and the radius is jointly determined by the principal-direction expansion result of the position prediction covariance and the set of beams where the user may be located. The principal direction of the covariance is used to characterize the direction in which the user position error expands most significantly. An uncertainty line segment is constructed along the principal direction of the covariance, and the beam set intersecting with the principal-direction uncertainty line segment is obtained. According to the maximum distance from the beam centers in this set to the location-area center, the radius of the circular dynamic location area is calculated, and the dynamic location area is then constructed.

For simplicity, the current posterior state estimate is denoted as ${\hat{x}}_{t|t}$ and the posterior covariance is denoted as $P_{t|t}$. The construction of the dynamic location area depends on the result of user-state prediction. The user-state prediction vector at time t is defined as ${\hat{x}}_{t}={\left[{\hat{r}}_{u,t}^{T},{\hat{v}}_{u,t}^{T},{\hat{a}}_{u,t}^{T},{\hat{b}}_{R,t}\right]}^{T}$, the state estimation error covariance matrix is denoted as $P_{t}$, and the position-related covariance submatrix is $P_{r,t}=P_{t}(1:3,1:3)$. The covariance matrix represents the uncertainty of the state prediction result. $P_{r,t}$ is used to analyze the principal expansion direction of the user position error. The dynamic location-area radius is jointly determined by the principal direction of this error distribution and the beam coverage structure.

At each time instant, the proposed TSM-SRCKF method completes model interaction, state prediction, measurement update, and model-probability fusion, obtaining the fused state estimate ${\hat{x}}_{t}$ and covariance matrix $P_{t}$. The dynamic location area takes the position component in the fused state as its center:

(32) $$c_{LA,t}={\hat{r}}_{u,t}$$

where $c_{LA,t}$ denotes the center of the dynamic location area at time t. Since the beam coverage at the user altitude can be represented on the local horizontal plane, the position covariance in the ECEF coordinate system needs to be transformed into the local ENU coordinate system at the user position.

Let ${\hat{\varphi }}_{u,t}$ and ${\hat{\lambda }}_{u,t}$ denote the latitude and longitude corresponding to the center of the dynamic location area, respectively. The rotation matrix from the ECEF coordinate system to the local ENU coordinate system is denoted as $R_{e2n,t}$. Then, the position covariance in the ENU coordinate system is

(33) $$P_{enu,t}=R_{e2n,t}P_{r,t}R_{e2n,t}^{T}$$

Let the horizontal two-dimensional position covariance be $P_{2,t}$, and we have $P_{2,t}=P_{\mathrm{enu},t}(1:2,1:2)$. Since the user position estimation error is usually not isotropic in the horizontal plane, the error at some time instants may mainly expand along the user’s flight direction, or may become more significant in a certain direction due to measurement geometry and filtering errors. To describe this directionality, we further perform eigenvalue decomposition on the horizontal two-dimensional covariance matrix $P_{2,k}$ and take the eigenvector corresponding to the largest eigenvalue as the principal expansion direction of the horizontal position error:

(34) $$P_{2,t}e_{1,t}=\lambda_{\max,t}e_{1,t}$$

where $e_{1,t}$ denotes the principal direction of the horizontal position error and $\lambda_{\max,t}$ denotes the error variance in this direction. The standard deviation along the principal direction is $\sigma_{\max,t}=\sqrt{\lambda_{\max,t}}$.

This direction only indicates the direction in which the error expands most significantly, and does not mean that there is no error in other directions. Uncertainty in other directions still exists. However, in the current method, separate regions are not constructed for other directions; instead, they are jointly covered by the final circular location area.

After obtaining the principal direction of the covariance, this paper constructs a finite-length uncertainty line segment along the principal direction $e_{1,t}$, with the dynamic location-area center $c_{LA,t}$ as the reference point. This line segment is used to describe the region where the user position error is most likely to expand. The half-length of the line segment is jointly determined by the principal-direction standard deviation, the confidence coefficient, the error amplification factor, and the fixed guard distance:

(35) $$L_{t}=\alpha n_{\sigma }\sigma_{\max,t}+d_{\mathrm{guard}}$$

where α is the error amplification factor, $n_{\sigma }$ is the confidence coefficient, and $d_{\mathrm{guard}}$ is the fixed guard distance. To avoid paging failure caused by an excessively short uncertainty line segment, or excessive redundant beams caused by an excessively long line segment, $L_{t}$ is constrained by upper and lower bounds:

(36) $$L_{t}=\min\left(\max\left(L_{t},L_{\min}\right),L_{\max}\right)$$

where $L_{\min}$ and $L_{\max}$ are the minimum and maximum half-lengths of the principal-direction uncertainty line segment, respectively. Let the number of discrete sampling points on the principal-direction line segment be $N_{l}$. Then, the principal-direction offset corresponding to the sampling point g is

(37) $$s_{g}=-L_{t}+\frac{2L_{t}(g-1)}{N_{l}-1},\;g=1,2,\ldots ,N_{l}$$

Therefore, the discrete sampling points on the principal-direction uncertainty line segment can be expressed as

(38) $$p_{t,g}=c_{\mathrm{LA},t}+s_{g}e_{1,t},\;g=1,2,\ldots ,N_{l}.$$

In the following, $p_{t,g},c_{\mathrm{LA},t}$ and $p_{b,i,j}(t)$ all denote two-dimensional coordinates projected onto the local ENU horizontal plane of the user.

Since paging is ultimately performed within satellite beams, the principal-direction uncertainty line segment needs to be mapped to the actual beam coverage set. Let spot beam j of satellite i be denoted as $B_{i,j,t}$, its beam center be $p_{b,i,j}(t)$, and its beam radius be $R_{\mathrm{eff},i}(t)$. If the distance from a beam center to any sampling point on the principal-direction line segment does not exceed the beam radius at the user altitude, then the beam is considered to intersect with the principal-direction uncertainty region. Therefore, the principal-direction candidate beam set is defined as

(39) $$S_{\mathrm{line},t}=\left\{B_{i,j,t}|\exists g,\;d\left(p_{t,g},p_{b,i,j}(t)\right)\leq R_{\mathrm{eff},i}(t)\right\}$$

where d(⋅,⋅) denotes the Euclidean distance between two points on the user’s local ENU horizontal plane. $S_{\mathrm{line},t}$ reflects the set of beams that may cover the user along the principal expansion direction of the user position error. If the principal-direction candidate beam set $S_{\mathrm{line},t}$ is nonempty, the maximum distance from the centers of these beams to the location-area center is calculated and combined with the corresponding effective beam radius at the user altitude, yielding the original dynamic location-area radius:

(40) $$R_{\mathrm{raw},t}=\max_{B_{i,j,t}\in S_{\mathrm{line},t}}[d\;\left(c_{\mathrm{LA},t},p_{b,i,j}(t)\right)+R_{\mathrm{eff},i}(t)]$$

To prevent the dynamic location-area radius from becoming excessively small or large, the raw radius $R_{\mathrm{raw},t}$ is constrained within the predefined lower and upper bounds $R_{\min}$ and $R_{\max}$, respectively, yielding the final dynamic location-area radius $R_{\mathrm{LA},t}$. Thus, the circular dynamic location area at time t can be expressed as

(41) $$L_{t}=\left\{r:d\;\left(r,c_{\mathrm{LA},t}\right)\leq R_{\mathrm{LA},t}\right\}$$

After the circular dynamic location area is determined, all beams located within the circular region are selected to form the paging beam set. This set is denoted as $S_{\mathrm{LA},t}$, and is defined as $S_{\mathrm{LA},t}=\left\{B_{i,j,t}|d\;\left(c_{\mathrm{LA},t},p_{b,i,j}(t)\right)\leq R_{\mathrm{LA},t}\right\}$. Here, at time t, $S_{\mathrm{LA},t}$ is the paging beam set delivered by the network side to the communication satellites. They only need to send paging messages within the spot beams corresponding to $S_{\mathrm{LA},t}$, and do not need to continuously maintain the complete motion state of the user during non-paging periods.

Let $N_{beam,t}=\left|S_{\mathrm{LA},t}\right|$ denote the number of beams participating in the paging process at time t. If the distance between the user’s true position and the center of a paging beam does not exceed its effective beam radius at the user altitude, this paging attempt is considered successful, which can be expressed as:

(42) $$I_{\mathrm{succ},t}=\left\{\begin{array}{ll} 1, & \exists B_{i,j,t}\in S_{t}^{LA},\;d\left(r_{u,t},P_{b,i,j}(t)\right)\leq R_{eff,i}(t),\\ 0, & \mathrm{otherwise}. \end{array}\right.$$

where $I_{\mathrm{succ},t}$ denotes the paging-success indicator at time t, with $I_{\mathrm{succ},t}=1$ indicating a successful paging attempt and $I_{\mathrm{succ},t}=0$ otherwise. Then, the paging success probability is defined as $P_{\mathrm{succ}}=\frac{N_{\mathrm{succ}}}{N_{page}}$, where $N_{succ}$ denotes the number of successful paging attempts and $N_{page}$ denotes the total number of paging attempts.

The system management overhead consists of the location-management signaling overhead and the paging overhead. Let a denote the unit signaling overhead associated with one location-management signaling event, and let b denote the unit signaling overhead incurred when one beam participates in a paging attempt. For the proposed method, one location-management signaling event corresponds to one paging-triggered location-area delivery, whereas for the TDLAM method, it corresponds to one location update. These two parameters are treated as fixed unit overheads in the simulations and are applied consistently to all compared methods to provide a unified basis for management-overhead evaluation. The paging overhead of the paging attempt t is $C_{page,t}=bN_{\mathrm{beam},t}$.

The cumulative total management overhead is $C_{\mathrm{total}}=C_{\mathrm{LUP}}+C_{\mathrm{page}}$, where $C_{LUP}$ is the cumulative overhead of location-area delivery or location update and $C_{page}$ is the cumulative paging overhead. The process of the proposed TSM-SRCKF-DLAM method is summarized as Algorithm 2. This algorithm combines the directionality of the user position estimation error with the satellite spot-beam coverage structure, enabling the dynamic location area to adaptively vary with the state estimation error and beam distribution. Compared with a fixed-radius location area, the proposed method can reduce unnecessary redundant paging beams. Compared with the traditional active location update mechanism, the paging-triggered delivery mechanism can reduce the signaling overhead caused by frequent location updates of high-mobility users.


**Algorithm 2:** Dynamic Location Area Design Algorithm Based on User Position and Its Covariance
**Input:**
$\mathrm{Fused}\;\mathrm{state}\;\mathrm{estimate}\;{\hat{x}}_{t}$ at time *t*, state estimation covariance matrix $P_{t},$ current satellite ephemeris and spot-beam center set, ground-projected spot-beam radius $R_{b},$ lower and upper bounds of the dynamic location area radius $R_{\min}$ $\mathrm{and}\;R_{\max}.$1Extract the user position estimate ${\hat{r}}_{u,t}$ $\mathrm{from}\;{\hat{x}}_{t},$ $\mathrm{and}\;\mathrm{set}\;c_{LA,t}={\hat{r}}_{u,t}.$
2Extract the position covariance submatrix from $P_{t},$ and we have $P_{r,t}=P_{t}(1:3,1:3).$
3$\mathrm{Transform}\;P_{r,t}$ into the user local ENU coordinate system to obtain $P_{enu,t}.$4Extract the horizontal two-dimensional covariance matrix $P_{2,t}=P_{\mathrm{enu},t}(1:2,1:2).$
5$\mathrm{Perform}\;\mathrm{eigenvalue}\;\mathrm{decomposition}\;\mathrm{on}\;P_{2,t},$ and obtain the largest eigenvalue $\lambda_{\max,t}$ $\mathrm{and}\;\mathrm{its}\;\mathrm{corresponding}\;\mathrm{eigenvector}\;e_{1,t}.$6Calculate the half-length $L_{t}$ of the principal-direction uncertainty line segment according to $\sigma_{\max,t}=\sqrt{\lambda_{\max,t}}.$7$\mathrm{Taking}\;c_{LA,t}$ as the center, construct the principal-direction uncertainty line segment along $e_{1,t},$ and perform discrete sampling.8Determine the effective beam radius $R_{\mathrm{eff},i}(t)$ and map the principal-direction sampling points onto the current spot-beam coverage to obtain the principal-direction candidate beam set $S_{\mathrm{line},t}.$
9Determine the original dynamic location-area radius $R_{\mathrm{raw},t}$ as the maximum, over all beams in $S_{\mathrm{line},t}$ of the sum of the distance from the beam center to the location-area center and the corresponding effective beam radius $R_{\mathrm{eff},i}(t).$
10Apply the upper and lower bound constraints to $R_{\mathrm{raw},t},$ and obtain the final dynamic location-area radius $R_{LA,t}.$
11Construct the circular dynamic location area $L_{t}$ according to $c_{LA,t}$ and $R_{LA,t}.$
12Select all spot beams located within the circular dynamic location area, and obtain the final paging beam set $S_{\mathrm{LA},t}.$
13When a paging request arrives, deliver $S_{\mathrm{LA},t}$ to the corresponding communication satellites, and the communication satellites send paging messages within the corresponding beams.
**Output**
$\mathrm{Dynamic}\;\mathrm{location}\;\mathrm{area}\;L_{t},$ final paging beam set $S_{\mathrm{LA},t}.$


## 5. Simulation Results and Performance Analysis

Referring to the article [19], the key simulation parameters are shown in Table 1.

Here, $g_{0}=9.81$ m/s^2^ denotes the standard gravitational acceleration. To further verify the effectiveness of the proposed high-mobility user state estimation method, the satellite tracking module is separately extracted from the processes of dynamic location area design, paging, and beam coverage management. Under the same high-mobility user trajectory, satellite visibility conditions, and ranging and Doppler measurement conditions, the estimation accuracy of the two methods is compared. To highlight the advantages of the proposed TSM-SRCKF method, the single constant-acceleration-model-aided cubature Kalman filtering (Single-CA-CKF) algorithm is selected as the comparison benchmark. This algorithm uses a fixed CA motion model to describe the user motion state and adopts the conventional CKF for nonlinear measurement update. It does not consider differences in the motion characteristics of high-mobility users during the ascent, cruise, and descent phases.

To comprehensively evaluate the user-state estimation performance, the three-dimensional position root-mean-square error, ${\mathrm{RMSE}}_{\mathrm{pos}}=\sqrt{\frac{1}{K}\sum_{t=1}^{K}{\left\|{\hat{r}}_{t}-r_{t}\right\|}^{2}}$ [20], is adopted as the primary evaluation metric, where K denotes the total number of sampling instants, while ${\hat{r}}_{t}$ and $r_{t}$ denote the estimated user position and the true user position at the t-th sampling instant, respectively. This metric measures the tracking accuracy by calculating the root-mean-square value of the three-dimensional position error over all sampling instants. A smaller ${\mathrm{RMSE}}_{\mathrm{pos}}$ indicates that the position estimation result is closer to the true trajectory, and, thus, the tracking performance is better.

**Figure 3:**
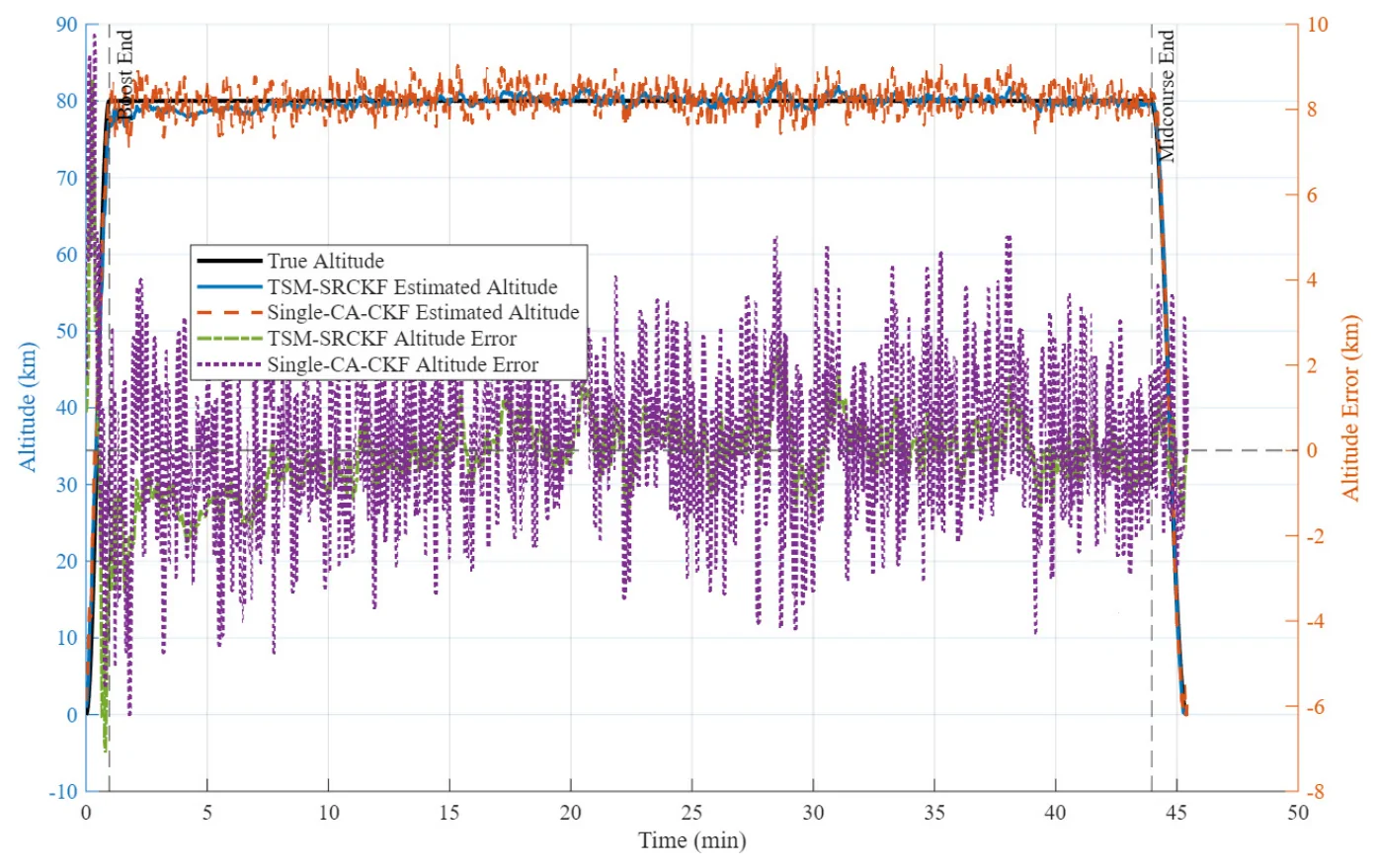
Comparison of user flight altitude and altitude error.

As shown in Figure 3, both methods can follow the overall altitude variation trend of the high-mobility user during ascent, cruise, and descent. However, the altitude error of the Single-CA-CKF algorithm fluctuates more significantly and shows obvious deviations at several time instants. The altitude estimated by the proposed method is closer to the true trajectory, indicating that multi-model fusion and adaptive measurement update can effectively alleviate the model mismatch problem during strong maneuvering phases.

**Figure 4:**
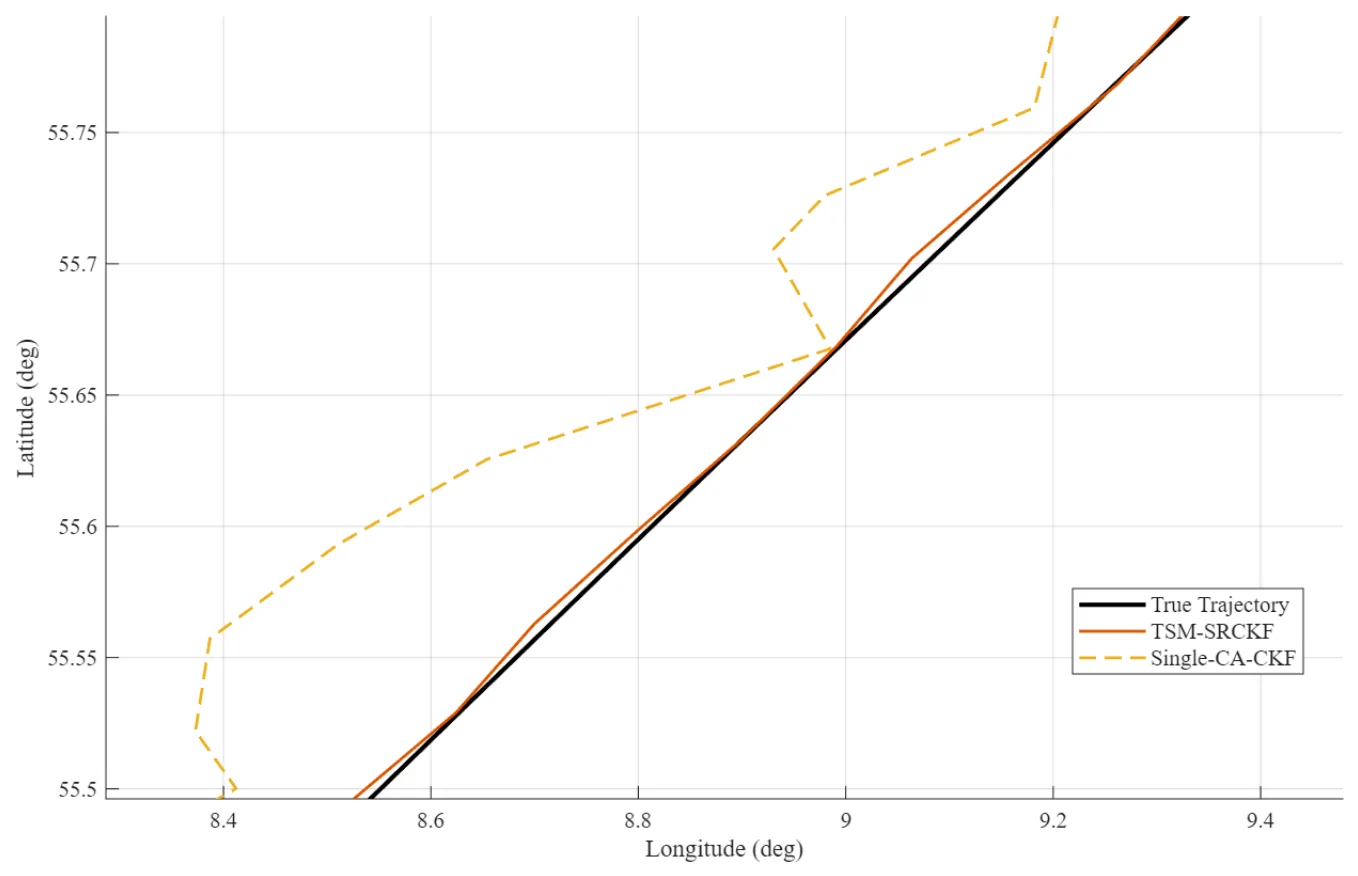
Longitude–latitude trajectories of both methods.

As shown in Figure 4, the longitude–latitude trajectories of both methods generally follow the true great-circle trajectory. However, the position error curve and altitude error curve show that the Single-CA-CKF algorithm exhibits more obvious three-dimensional deviations at local time instants. The ranging bias estimation result further indicates that the proposed method provides a more stable estimation of the bias state, which is beneficial for reducing the long-term influence of ranging system errors on position solution.

**Figure 5:**
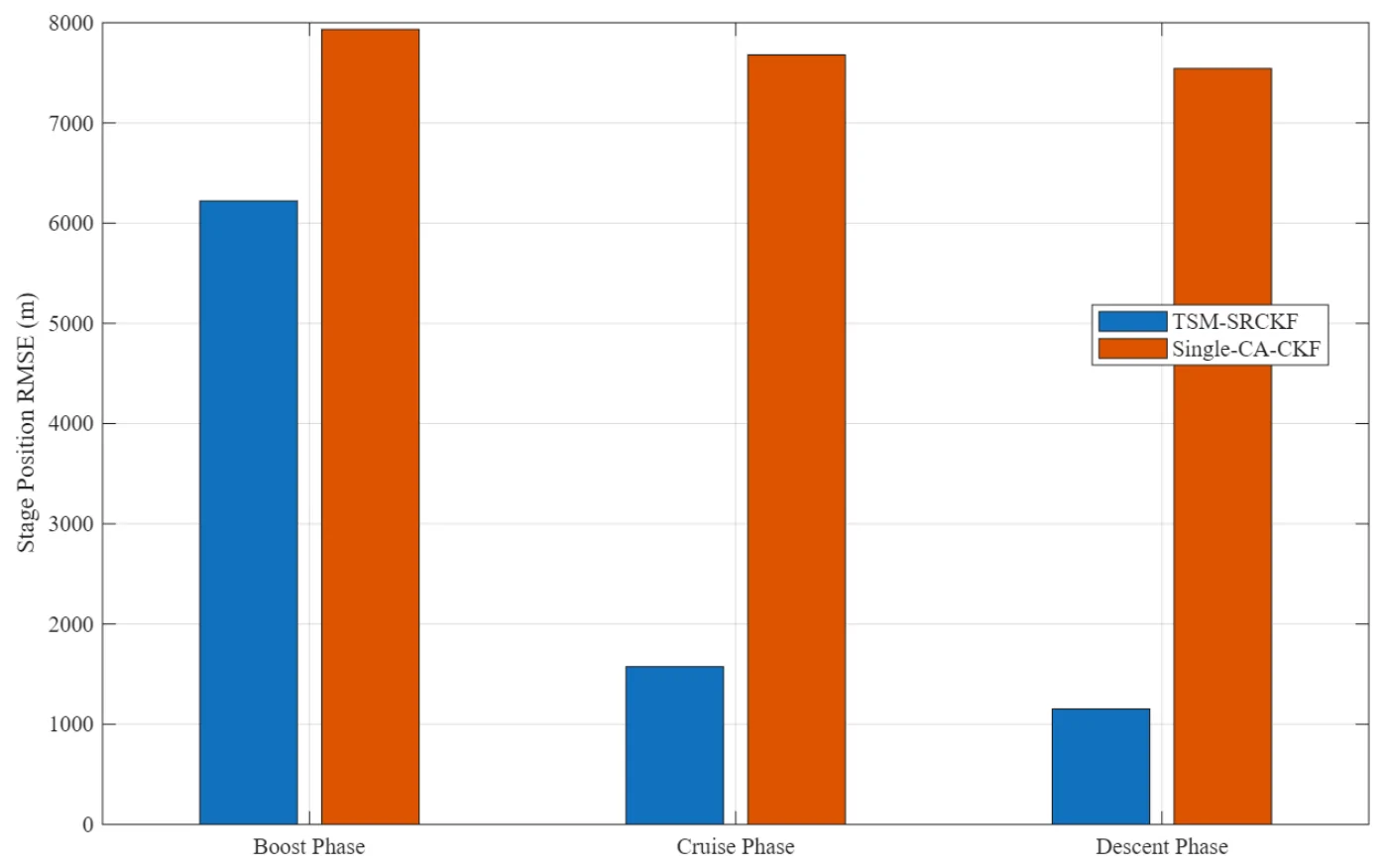
Comparison of RMSE of three stages.

As can be seen from Figure 5, the proposed method achieves significant advantages over the conventional Single-CA-CKF algorithm both in the cruise and descent phases. The error in the ascent phase is relatively large, which is mainly related to the initial filter convergence process, the strong acceleration during ascent, and the rapid variation in the radar cross-section (RCS). Overall, the simulation results show that the proposed method can effectively reduce the position prediction error under the three-stage motion conditions of high-mobility users and provide more reliable state prediction results for the proposed method of dynamic location areas.

**Figure 6:**
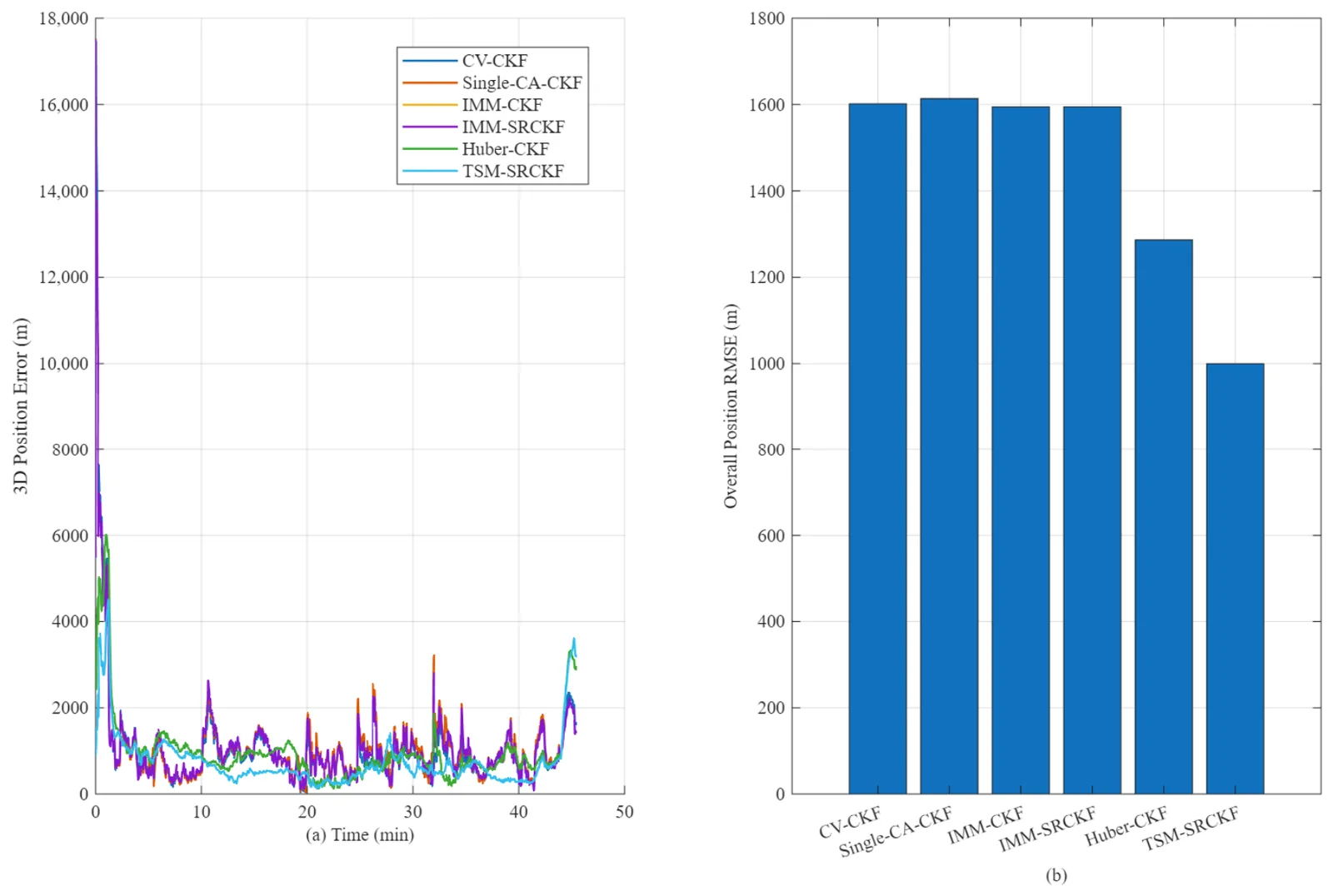
(**a**) 3D Position Error versus Time. (**b**) Overall Position RMSE of Different Filters.

To further evaluate the state-estimation performance, the proposed TSM-SRCKF method is compared with CV-CKF, Single-CA-CKF, IMM-CKF, IMM-SRCKF, and Huber-CKF under the same simulation conditions. As shown in Figure 6, TSM-SRCKF achieves the lowest overall position RMSE among the compared methods, demonstrating its improved state-estimation accuracy for high-mobility users.

**Figure 7:**
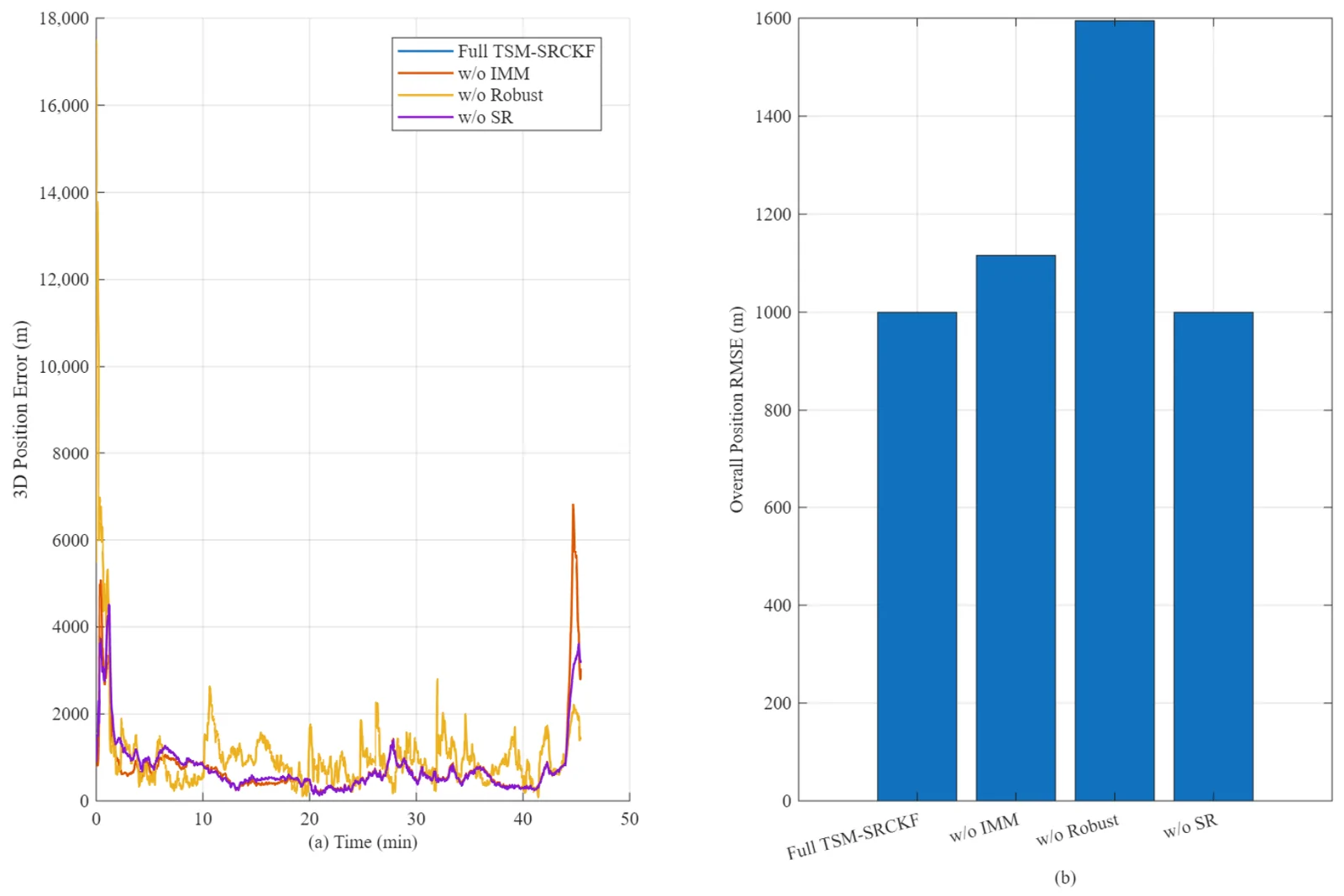
(**a**) Time-Varying 3D Position Error in the Ablation Study. (**b**) Overall Position RMSE in the Ablation Study.

To further analyze the contributions of the main components of TSM-SRCKF, an ablation study is conducted by removing the IMM mechanism, robust measurement update, and square-root implementation, respectively. As shown in Figure 7, removing the robust measurement update leads to the largest increase in position RMSE, while removing the IMM mechanism also degrades the estimation accuracy. The result without the square-root implementation exhibits a similar average RMSE to the full method, indicating that its main contribution lies in improving numerical stability rather than directly reducing the average estimation error.

**Figure 8:**
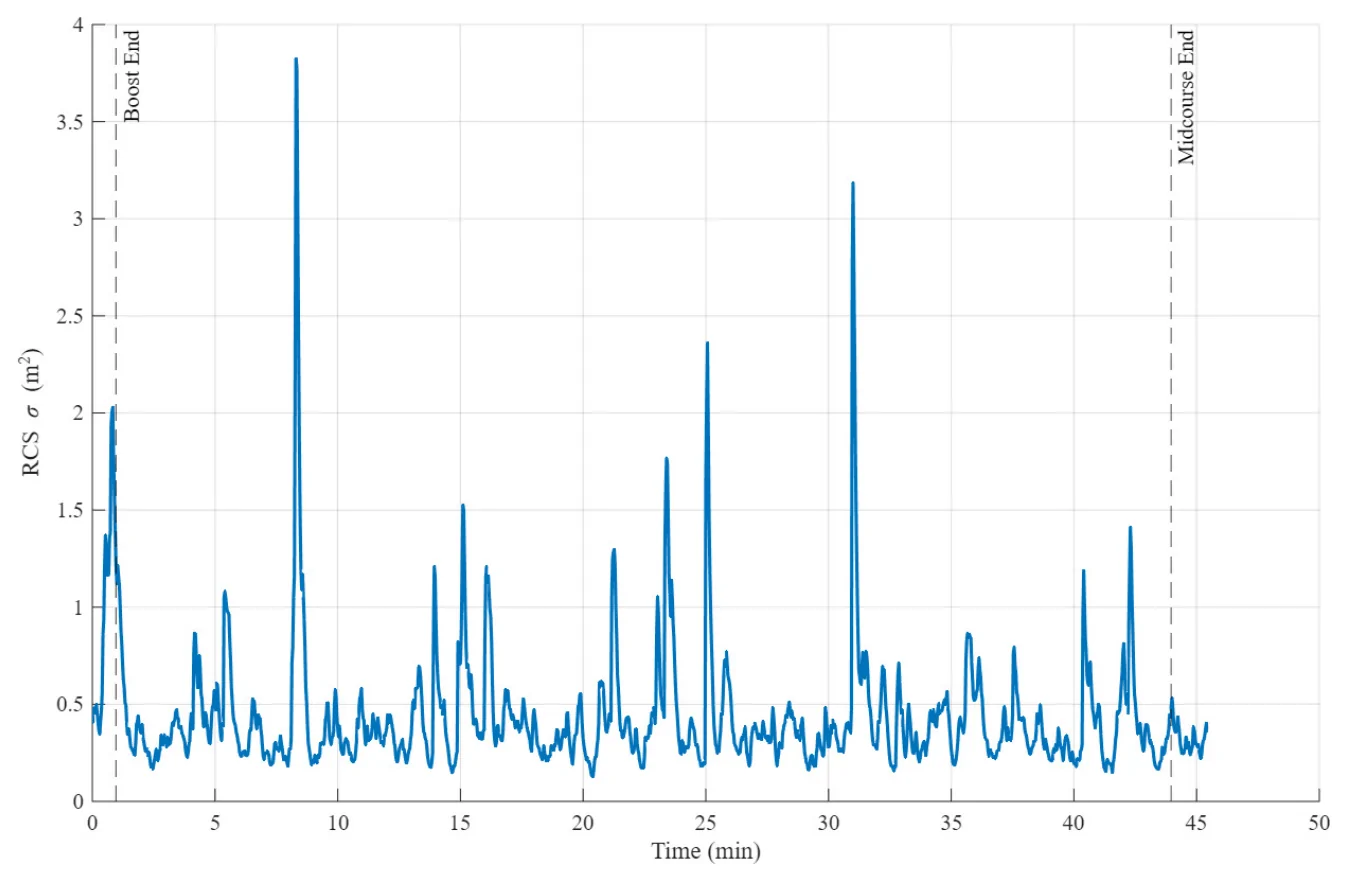
RCS of a high-mobility user versus flight time.

In addition, as shown in Figure 8, the variation in the user’s RCS with flight time is presented. It can be observed that the user’s RCS does not remain constant during flight, but exhibits significant fluctuations due to factors such as attitude variation, flight phase transition, and glint-induced strong scattering. At some time instants, peaks appear in the RCS curve, indicating stronger echoes from the user. In low-RCS regions, radar measurements are more susceptible to noise. This result demonstrates the obvious time-varying characteristics and uncertainty of satellite sensing measurements, and further verifies the necessity of the proposed method.

To further verify the effectiveness of the proposed TSM-SRCKF-DLAM method, a traditional dynamic location-area management (TDLAM) method is used for comparison purposes. The TDLAM method considered estimates the possible displacement of the user within the registration time scale according to the user’s horizontal velocity, and uses this movement distance as a reference for radius design. The higher the user velocity, the larger the traditional location area radius becomes. If the movement distance between the current predicted position and the previous registration center satisfies $d_{\mathrm{move}}\geq \beta R_{\mathrm{active}}$, or if the elapsed time since the previous location update exceeds the forced-update interval $T_{\mathrm{force}}$, the traditional location area design immediately performs a location update, where $d_{move}$ denotes the user movement distance, $R_{active}$ denotes the current dynamic location-area radius, β denotes the location-update triggering coefficient, and $T_{\mathrm{force}}$ denotes the maximum allowable interval between two consecutive location updates in the TDLAM method.

For the performance evaluation of dynamic location-area management, the paging success probability, the average number of paging beams per paging attempt, the number of location-management signaling events, the cumulative location-management signaling overhead, the total paging overhead, and the total management overhead are adopted as evaluation metrics. The average number of paging beams per paging attempt is defined as [21] ${\bar{N}}_{\mathrm{beam}}=\frac{1}{N_{\mathrm{page}}}\sum_{p=1}^{N_{\mathrm{page}}}N_{\mathrm{beam},t_{p}}$, where $N_{\mathrm{page}}$ denotes the total number of paging attempts, p denotes the paging-attempt index, $t_{p}$ denotes the time index corresponding to the p-th paging attempt, and $N_{beam,t_{p}}$ denotes the number of beams participating in the p-th paging attempt. The total management overhead is defined as $C_{\mathrm{total}}=C_{LUP}+C_{\mathrm{page}}$, where $C_{LUP}$ denotes the cumulative location-management signaling overhead and $C_{\mathrm{page}}$ denotes the cumulative paging overhead.

In addition to the location-management signaling overhead and paging overhead, the overheads associated with satellite sensing, measurement reporting, inter-satellite forwarding, and network-side state estimation are also considered. The cumulative measurement-reporting overhead and inter-satellite forwarding overhead are defined as

$$C_{\mathrm{meas}}=b_{\mathrm{meas}}\sum_{t=1}^{K}M_{t},$$

$$C_{\mathrm{ISL}}=b_{\mathrm{meas}}{\bar{H}}_{\mathrm{ISL}}\sum_{t=1}^{K}M_{t}.$$

respectively, where $b_{means}$ denotes the number of bits contained in one measurement report and ${\bar{H}}_{\mathrm{ISL}}$ denotes the average number of inter-satellite forwarding hops. Accordingly, the cumulative system signaling overhead is defined as

$$C_{\mathrm{sig}}=C_{\mathrm{total}}+C_{\mathrm{meas}}+C_{\mathrm{ISL}}.$$

In addition, the satellite sensing overhead and state-estimation overhead are characterized by the cumulative sensing energy consumption and computation time, respectively, where $T_{\mathrm{est},t}$ denotes the computation time required for state estimation at sampling instant t. Since signaling overhead, sensing energy consumption, and computation time have different physical units, they are evaluated separately rather than directly combined into a single overhead metric.

**Figure 9:**
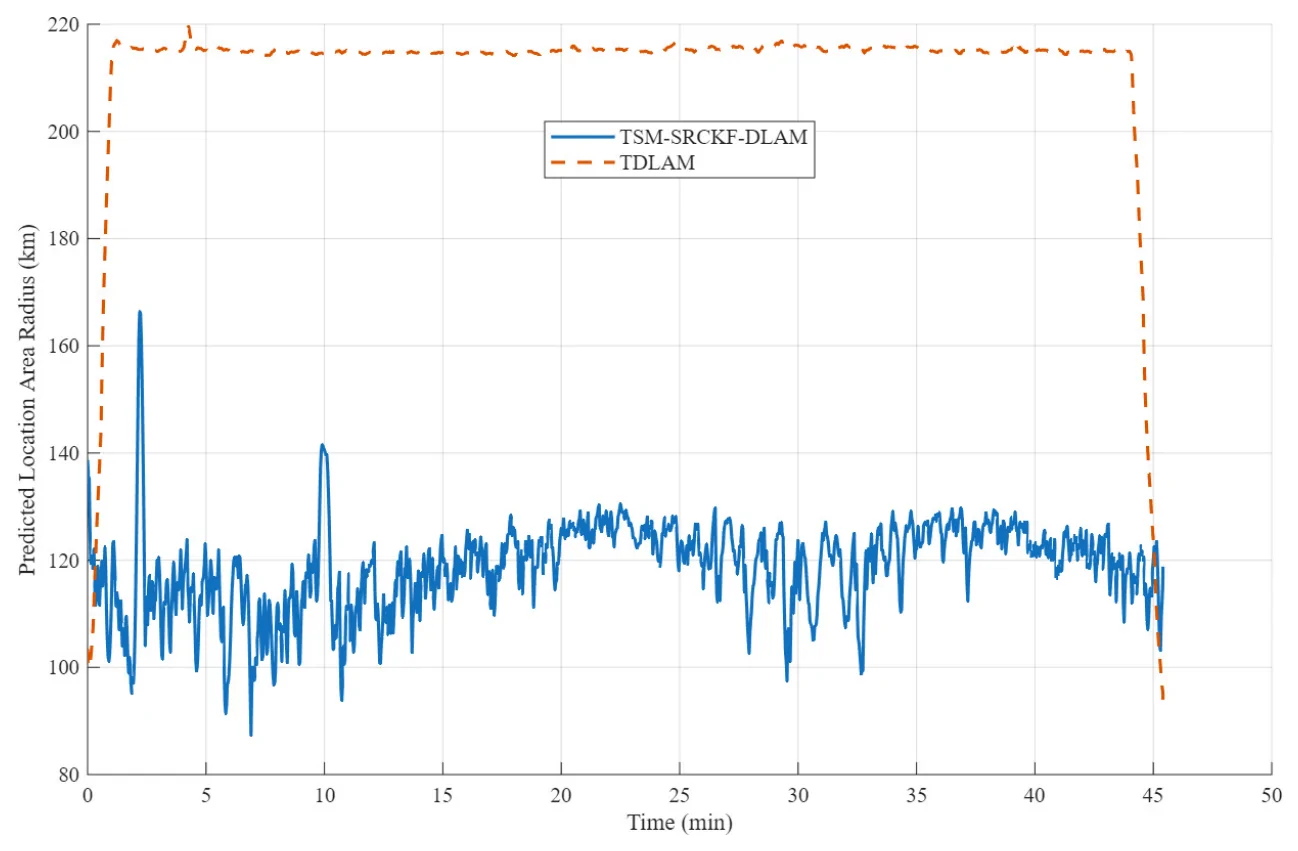
Dynamic location-area radii of the TSM-SRCKF-DLAM and TDLAM methods.

As can be seen from Figure 9, the predicted location-area radius of the traditional method considered remains above approximately 210 km for a long period, whereas the radius of the proposed TSM-SRCKF-DLAM method spans approximately from 100 km to 130 km. This is because the TDLAM method, using a larger circular location area, performs registration only according to the velocity and distance threshold. Additionally, the TDLAM method considered may occupy some beams that are unrelated to the actual possible region of the high-mobility user.

Table 2 and Table 3 show the principal-direction candidate beam sets and paging beam sets at the paging instants, respectively. In these tables, the labels “S” and “B” identify the satellite and spot beam, respectively.

**Figure 10:**
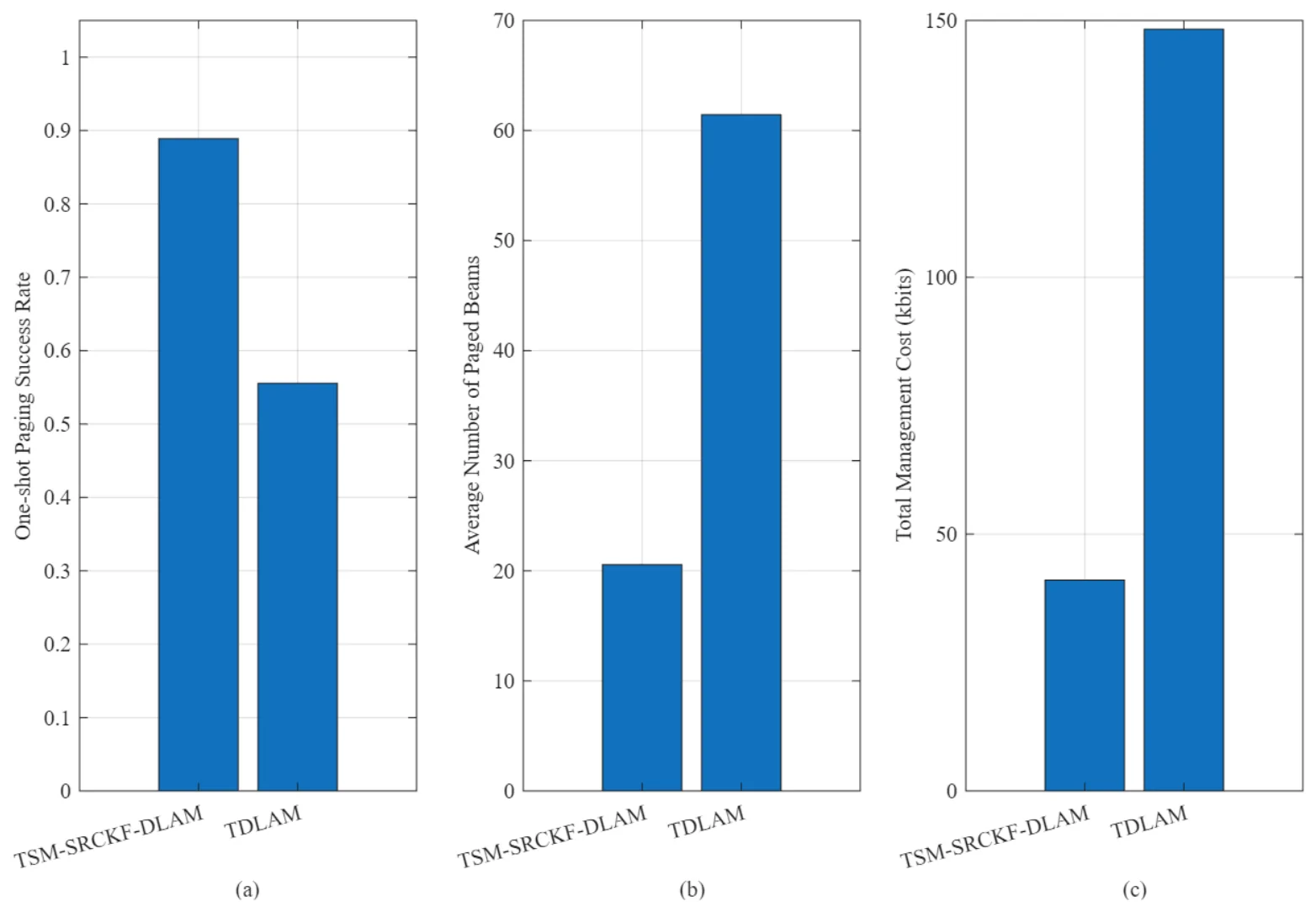
(**a**) One-Shot Paging Success Rate. (**b**) Average Number of Paged Beams. (**c**) Total Management Cost.

As shown in Figure 10a, the paging success probability of the TSM-SRCKF-DLAM method is approximately 0.889, whereas that of the TDLAM method is approximately 0.556. Compared with the traditional method, the proposed method provides higher paging reliability. Figure 10b presents the comparison of the average number of paging beams per paging attempt. It can be observed that the proposed method requires approximately 20 beams on average for each paging attempt, while the traditional method requires approximately 61 beams. The traditional method mainly constructs a circular location area based on a distance threshold, which makes it difficult to reflect the directionality of the prediction error and the spatial distribution relationship of satellite beams. Therefore, it tends to introduce more redundant beams. In contrast, the proposed method generates the user uncertainty region according to the principal direction of the covariance and maps it to the candidate beam set, thereby making the paging range more concentrated and reducing the participation of ineffective beams. Therefore, while ensuring a higher paging success probability, the proposed method significantly reduces the average number of paging beams per paging attempt.

Figure 10c presents the comparison of the total management overhead. Specifically, the total management overhead of the proposed method is approximately 40 kbits, whereas that of the traditional method is approximately 148 kbits, corresponding to an overhead reduction of about 73%. The total management overhead consists of the location-management signaling overhead and the paging overhead. The traditional method requires more frequent location updates and a larger number of paging beams, resulting in relatively high location-management signaling and paging overhead.

In contrast, the proposed method adopts a paging-triggered location-area delivery mechanism, in which the dynamic location area is constructed and delivered when a paging request occurs, thereby avoiding unnecessary location updates during non-paging periods. Meanwhile, the proposed method constructs the dynamic location area according to the user-state prediction, prediction uncertainty, and satellite beam geometry, thereby reducing redundant paging beams. Through the joint reduction in location-management signaling overhead and paging overhead, the proposed method effectively decreases the total management overhead. Overall, Figure 10a–c demonstrate that the proposed dynamic location-area method improves the paging success probability, reduces the average number of paging beams, and significantly lowers the total management overhead.

To further distinguish the performance gains brought by the dynamic location-area construction method from those introduced by the location-management signaling mechanism, a controlled ablation experiment is conducted by combining two location-area construction methods with two signaling mechanisms. Specifically, four combinations are considered: Proposed + Paging-Triggered, Proposed + Threshold-Based, Traditional + Paging-Triggered, and Traditional + Threshold-Based. The former two combinations adopt the proposed covariance- and beam-geometry-based location-area construction method, whereas the latter two adopt the traditional velocity-based location-area construction method. For each location-area construction method, both the paging-triggered delivery mechanism and the threshold-based location-update mechanism are evaluated under the same simulation conditions.

**Figure 11:**
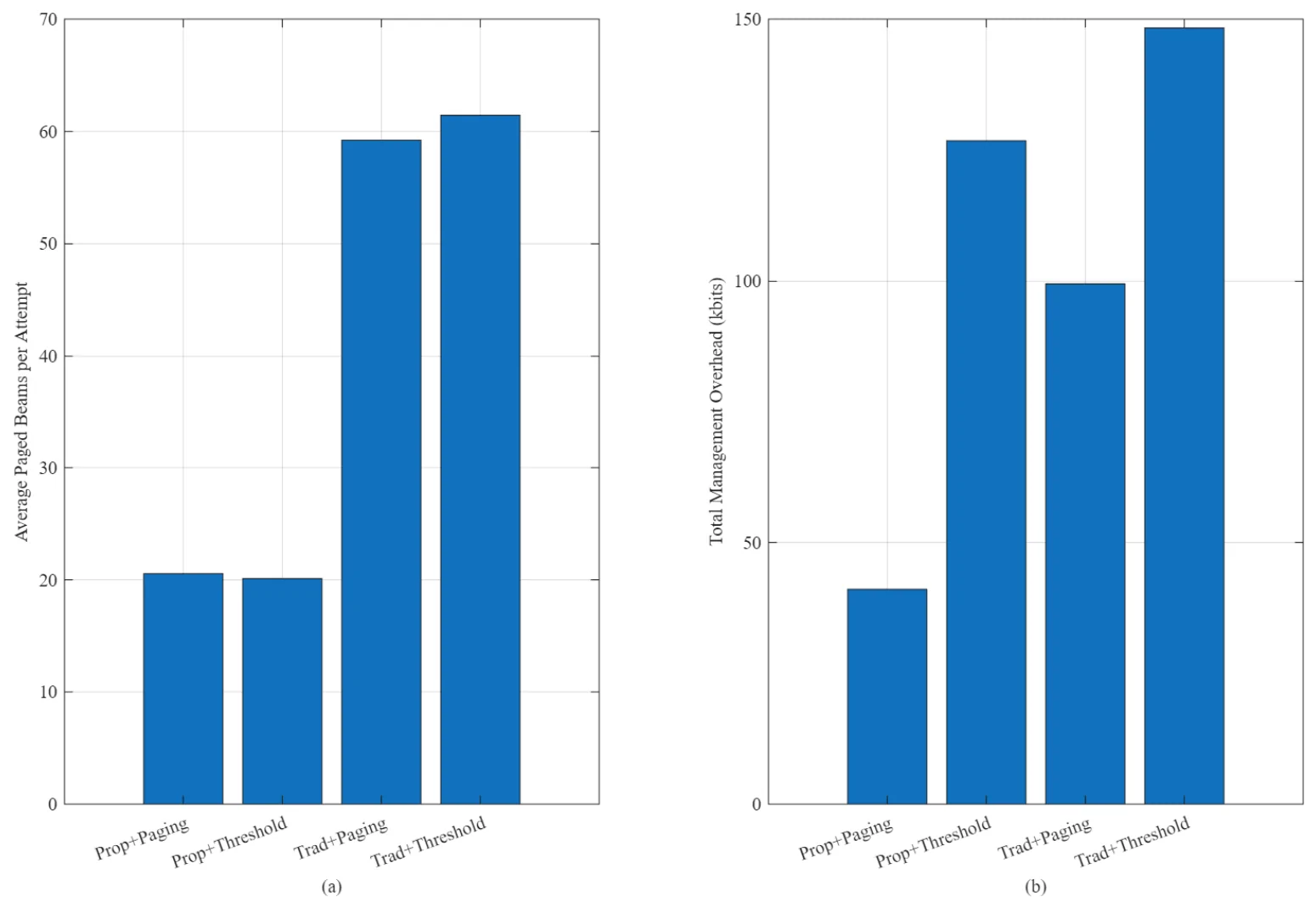
(**a**) Average Number of Paged Beams per Attempt. (**b**) Total Management Overhead.

As shown in Figure 11a, the proposed method requires about 20 paging beams per attempt, compared with about 59–61 beams for the traditional method, indicating that the proposed location-area construction effectively reduces redundant paging beams. Figure 11b further shows that the paging-triggered mechanism reduces the total management overhead under both location-area construction methods. Therefore, the overall overhead reduction results jointly from the proposed location-area construction method and the paging-triggered delivery mechanism. In addition to the management overhead evaluated above, the overheads associated with the complete sensing-assisted location-management process are further evaluated. Over the entire simulation period, the cumulative measurement-reporting overhead and inter-satellite forwarding overhead are 1353.088 kbits and 2706.176 kbits, respectively, while the cumulative satellite sensing energy consumption is 1.364 kJ. The state-estimation computation time is also recorded during the simulation and reported separately. Since all four controlled schemes employ the same satellite sensing measurements and state-estimation process, the measurement-reporting, inter-satellite forwarding, sensing, and state-estimation overheads are common to the compared schemes. Therefore, these common components increase the absolute system-level overhead but do not affect the relative management-overhead difference among the compared location-area construction and signaling mechanisms.

## 6. Conclusions

To address the issues of the mobility of high-mobility users and the dynamic space-ground topology in LEO satellite communication systems, this paper proposed a dynamic location area design method for a high-mobility user based on real-time detection and prediction by LEO satellites. First, the high-mobility user state estimation method integrating the three-stage motion model and square-root cubature Kalman filtering was used to estimate the motion state of the high-mobility user, thereby obtaining the predicted future position of the user and its prediction error covariance. Then, the principal expansion direction of prediction error was extracted via the covariance matrix to construct the user’s potential position interval. Furthermore, candidate beams, covering this range, were selected by considering the satellite beam coverage relationship. The predicted position was taken as the center of the dynamic location area, thereby constructing a circular dynamic location area and determining the beam set that needs to participate in paging. The simulation results show that, compared with Single-CA-CKF, the proposed TSM-SRCKF method reduces the position tracking RMSE in the ascent, cruise, and descent phases by approximately 21.5%, 79.7%, and 85.0%, respectively. Compared with the traditional location area design method, the paging success probability can be improved by approximately 60.1%, demonstrating that the proposed method can effectively enhance paging reliability.

## Figures and Tables

**Table 1:** Key simulation parameters.

L	30	G	60
$R_{e}$	6371 km	$h_{s}$	508 km
$v_{u,\max}$	Mach10	$a_{u,\mathrm{up}}$	$5\;g_{0}$
$a_{u,\mathrm{down}}$	$-4\;g_{0}$	$h_{u,c}$	80 km
$N_{s}$	1800	$N_{b}$	60
$R_{b}$	45 km	$P_{tx}$	100 W
$f_{c}$	1 GHz	λ	0.3 m
$N_{p}$	50	$T_{p}$	50 μs
$T_{\mathrm{coh}}$	0.2 s	$T_{\mathrm{idle}}$	1.8 s
a	1108 bits	b	168 bits
Initial latitude and longitude	[31.72, 118.88]	Destination latitude and longitude	[51.50, −0.127]
$B_{LFM}$	500 kHz	$G_{tx}$	30 dB
$G_{rx}$	25 dB	$F_{n}$	3 dB
$B_{n}$	1 kHz	$L_{sys}$	6 dB
$T_{0}$	290 K	$M_{\max}$	4

**Table 2:** Principal-direction candidate beam sets at paging instants.

Paging Time/min	Set of Candidate Beams in the Principal Direction
5	S0290−B43,S0290−B52,S1385−B40
10	S0170−B48
15	S0108−B20,S0108−B38,S0108−B44,S1496−B26,S1496−B35
25	S1488−B19,S1488−B30,S1488−B37,S1488−B53,S1546−B40, S1546−B50,S1606−B07,S1606−B11,S1606−B19,S1606−B37
30	S1424−B36,S1424−B52,S1484−B41
45	S1000−B32,S1000−B56,S1059−B45,S1471−B29,S1471−B30, S1471−B37

**Table 3:** Paging beam sets at paging instants.

Paging Time/min	Paging Beam Set
5	S0290−B29,S0290−B36,S0290−B43,S0290−B52,S0290−B59, S1385−B22,S1385−B40,S1385−B50
10	S0170−B35,S0170−B47,S0170−B48,S1381−B51,S1381−B59
15	S0108−B20,S0108−B24,S0108−B33,S0108−B38,S0108−B44, S0108−B45,S0108−B57,S1496−B09,S1496−B12,S1496−B17, S1496−B25,S1496−B26,S1496−B27,S1496−B35,S1496−B42, S1496−B47,S1496−B48
25	S1488−B07,S1488−B11,S1488−B13,S1488−B19,S1488−B29, S1488−B30,S1488−B31,S1488−B37,S1488−B43,S1488−B53, S1488−B54,S1488−B60,S1546−B22,S1546−B28,S1546−B32, S1546−B40,S1546−B50,S1546−B56,S1606−B07,S1606−B11, S1606−B13,S1606−B19,S1606−B23,S1606−B30,S1606−B31, S1606−B37
30	S1424−B18,S1424−B28,S1424−B29,S1424−B30,S1424−B36, S1424−B43,S1424−B51,S1424−B52,S1424−B59,S1484−B41, S1484−B54,S1484−B60,S1542−B43,S1542−B52
45	S1000−B32,S1000−B49,S1000−B50,S1000−B56,S1059−B24, S1059−B38,S1059−B44,S1059−B45,S1471−B13,S1471−B19, S1471−B29,S1471−B30,S1471−B37,S1471−B43,S1471−B53, S1471−B60

## Data Availability

Data is contained within the article. The original contributions presented in this study are included in the article. Further inquiries can be directed to the corresponding author.
